# Unmanned aerial vehicle payload technology applications in agriculture and other low-altitude scenarios: a review

**DOI:** 10.3389/fpls.2025.1721484

**Published:** 2025-12-12

**Authors:** Weixiang Yao, Changliang Liu, Yuzhou Liu, Qi Zheng, Junyong Wang, Huiduo Yu, Chunling Chen, Shuang Guo

**Affiliations:** 1 College of Information and Electrical Engineering, Shenyang Agricultural University, National Digital Agriculture Regional Innovation Center (Northeast), Shenyang, China; 2 School of Intelligent Science and Information Engineering, Shenyang University, Shenyang, China

**Keywords:** agriculture, UAV, payload technology, application scenarios, low-altitude economy

## Abstract

Unmanned Aerial Vehicle (UAV), as a new generation of intelligent equipment, has gradually become an essential tool across multiple industries due to its high maneuverability and strong task adaptability. UAV payload technology (UPT) serves as a key support for enhancing mission performance and expanding application scenarios. UPT is being rapidly integrated into agriculture and other key fields, emerging as a driving force for the low-altitude economy and intelligent operations. This study systematically analyzed and discussed the development status of UPT, its typical application scenarios, and the challenges faced. By conducting a comprehensive review of global research on UPT from 2012 to 2025, this review summarized research hotspots and revealed evolutionary trends. The findings demonstrated that UPT had made notable progress in typical application areas, including crop monitoring, precision agricultural operations, agricultural product harvesting and aerial transportation, power line inspection, emergency rescue, and logistics. However, UPT was still constrained by limited autonomous perception and path planning capabilities, insufficient universality of payload platforms, a lack of standardized device interfaces, as well as challenges related to endurance, communication, and operational stability under adverse weather conditions. Future research should focus on lightweight and multifunctional payload design, intelligent operation control, and modular and standardized integration, while building a “satellite-UAV-ground” collaborative perception and decision-making system. The outcomes of this study provide both theoretical reference and practical guidance for promoting UAV adoption in agriculture and other low-altitude application scenarios, thereby contributing to the sustainable development of smart agriculture and the low-altitude economy.

## 1 Introduction

In recent years, with the continuous maturation of unmanned aerial vehicle (UAV) technology, unmanned aerial vehicles (UAVs) have been evolving toward platformization (He et al., 2024), modularization (Zhang et al., 2023), and intelligentization (Li et al., 2025c). As the core operational component of UAV missions, mission payloads have increasingly become a focal point of research in low-altitude aerial systems (Ariante and Core, 2025). A UAV’s payload serves as the central device for completing various tasks, directly influencing its operational capabilities and range of applications. It should be noted that there is a fundamental conceptual distinction between the “UAV payload,” which refers to specific execution devices, and “UAV payload technology (UPT),” a comprehensive technical system. The former focuses on the realization of physical functions, while the latter emphasizes payload integration, control, and system coordination. UAV payload encompasses multiple disciplines, including agricultural engineering, aeronautical engineering, automatic control, artificial intelligence, and materials science. Its primary functions include data acquisition, precision agriculture, and material transport, making it a critical component of UPT in agricultural and other low-altitude application scenarios (Ayamga et al., 2021).

UAV mission payloads are diverse and typically include sensors-such as visible-light, infrared, multispectral, and LiDAR sensors-and actuation devices, such as spraying systems, gripping mechanisms, and hoisting modules. They encompass key technologies for high-resolution imaging, multimodal perception, and task execution. From a performance perspective, different types of payloads exhibit significant differences. Sensor payloads are generally lightweight and energy-efficient but relatively costly, primarily serving data acquisition and environmental monitoring purposes. Actuation payloads, such as gripping or spraying modules, have moderate weight and higher power consumption, directly performing operational tasks, but they impose greater demands on UAV endurance and flight stability (Chen et al., 2023). Overall, sensor and actuation payloads offer distinct advantages in weight, power consumption, cost, and application scenarios, enabling UAVs to meet diverse operational needs. In agricultural practice, imaging payloads are widely used for precision crop monitoring and protection (He et al., 2025). Spraying systems and variable-rate control units have played a crucial role in promoting more intelligent farming operations (Song et al., 2021; Wang et al., 2022). With these technical foundations, UAV payloads have become central to agricultural applications. Platforms equipped with multispectral and hyperspectral sensors or LiDAR have been employed for crop growth assessment (Fan et al., 2023; Lian et al., 2024), yield prediction (S and K, 2023; Sekharamantry et al., 2024; Zhao et al., 2024a), pest and disease detection (Yang et al., 2025), precision fertilization (Gheorghe et al., 2024), and variable-rate spraying (Taseer and Han, 2024), significantly advancing the precision and automation of farm management. Beyond agriculture, UAV payloads also find applications in industrial inspection and maintenance (Ayele et al., 2020; Ling et al., 2025), military reconnaissance and explosive ordnance disposal (Nikulin et al., 2018; Lidong et al., 2023), and environmental monitoring (Liang and Shen, 2023; Klopfenstein and Lussier Desbiens, 2024; Yuting and Jinrong, 2024), contributing to infrastructure safety, situational awareness, and ecological conservation.

In the context of the rapidly expanding global low-altitude economy, mission payloads are recognized as a key component in completing the “perception-decision-execution” cycle. They also provide the technical foundation for establishing a low-altitude airspace management system that is reliable, controllable, and easily governed (Ali et al., 2022; Yao et al., 2022). Nevertheless, challenges such as limited endurance, inefficient data processing, and the lack of unified interface standards continue to hinder the stable performance and large-scale application of mission payloads (Zhang et al., 2022). Against this backdrop, an in-depth examination of payload architectures and their adaptability across industries is essential to driving the intelligent upgrading of drones and accelerating the industrialization of low-altitude operations.

This paper provides a systematic review of global research on UAV payload technology from 2012 to 2025. Conducting statistical analyses of core keywords, research hotspots, and technological evolution, it uncovers the major developmental trends in the field. Building on these findings, the study outlines the current state of UPT in agriculture and other representative low-altitude applications, highlights key technological bottlenecks that demand breakthroughs, and discusses potential directions for future advancement. The aim is to help researchers gain a clearer understanding of the developmental trajectory of UPT, thereby offering valuable references and insights for both academic research and practical applications.

## 2 Review methodology

**Figure 1:**
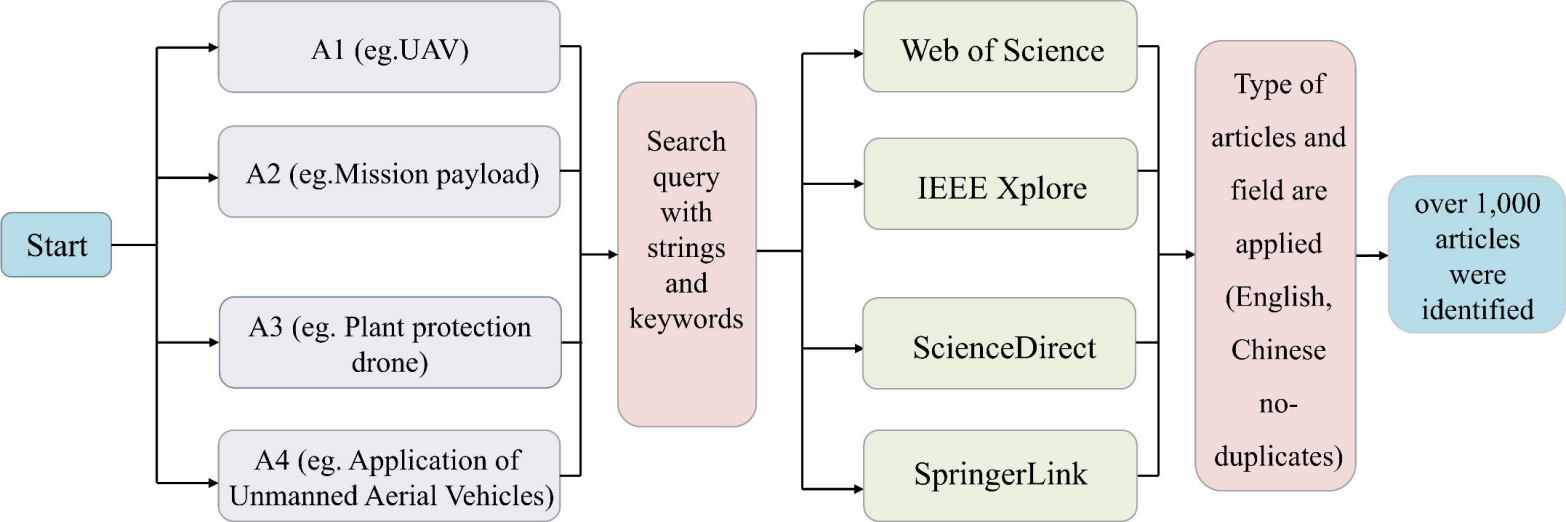
Literature search methodology.

To comprehensively examine the development trajectory of UAV mission payload technologies and their application trends in agriculture and other typical scenarios, this study systematically reviewed global research findings published between 2012 and 2025. The literature was primarily sourced from major international databases, including Web of Science, IEEE Xplore, ScienceDirect, SpringerLink, Elsevier, and the SciELO Citation Index, as well as authoritative Chinese platforms such as China National Knowledge Infrastructure (CNKI) and Wanfang Data, encompassing a wide range of high-quality studies. Search keywords included “Unmanned Aerial Vehicle (UAV)”, “Unmanned Aircraft System”, “Application of Unmanned Aerial Vehicles”, “Mission Payload”, “Modular Load System”, and “Plant Protection Drone”, covering both technical perspectives and application contexts. An initial screening yielded more than 1,000 documents, from which over 190 papers were ultimately selected as the theoretical foundation based on criteria such as thematic relevance, technical depth, and citation frequency. The detailed search methodology is illustrated in **Figure 1**.

**Figure 2:**
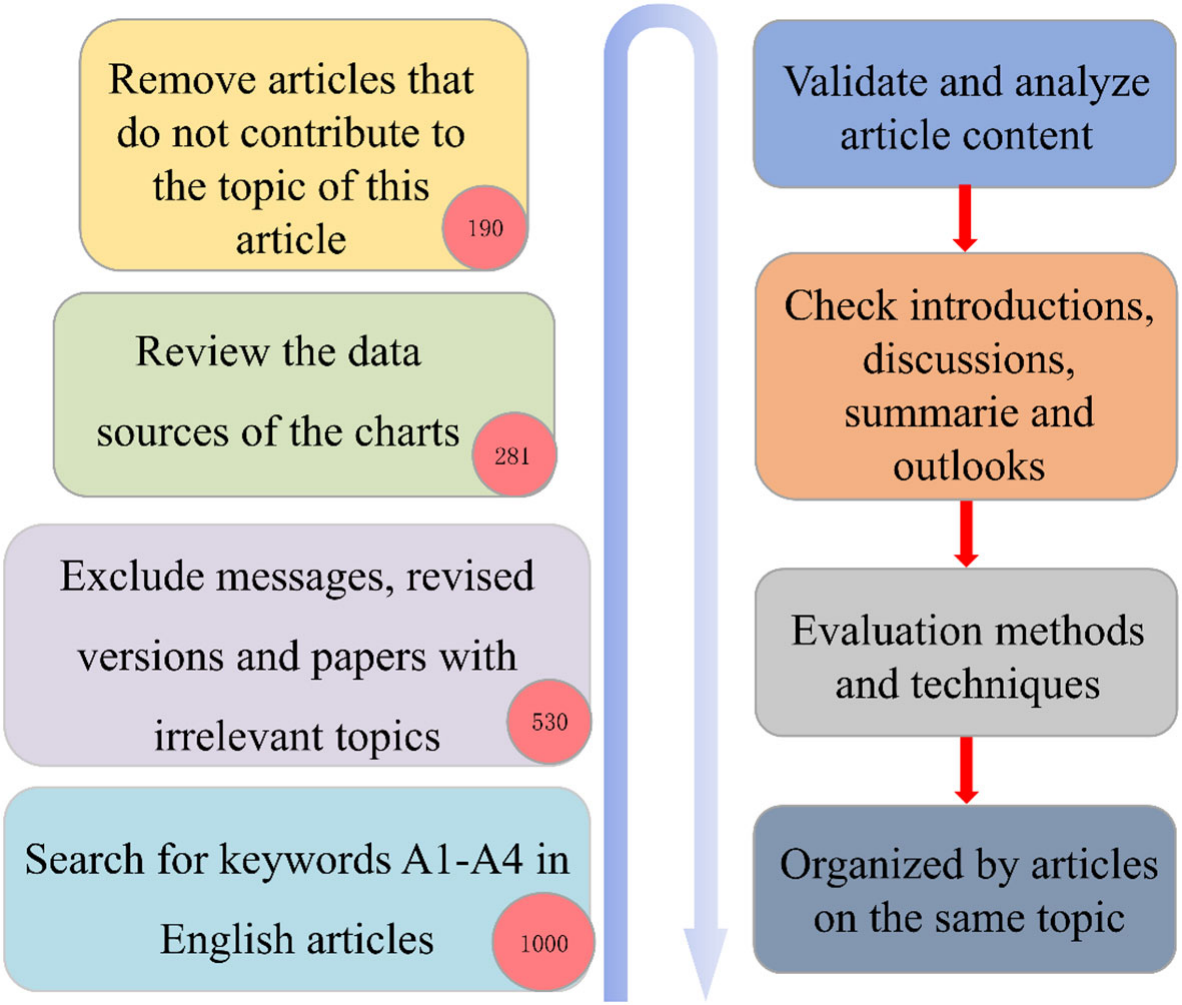
Literature review methodology.

This study adopted a systematic screening strategy to exclude newsletters, preliminary drafts, and irrelevant publications. The review emphasized the examination of chart data, dataset sources, and technical relevance, while also evaluating the feasibility of algorithms and their application value. Ultimately, representative studies were retained. The detailed workflow is presented in **Figure 2**.

**Figure 3:**
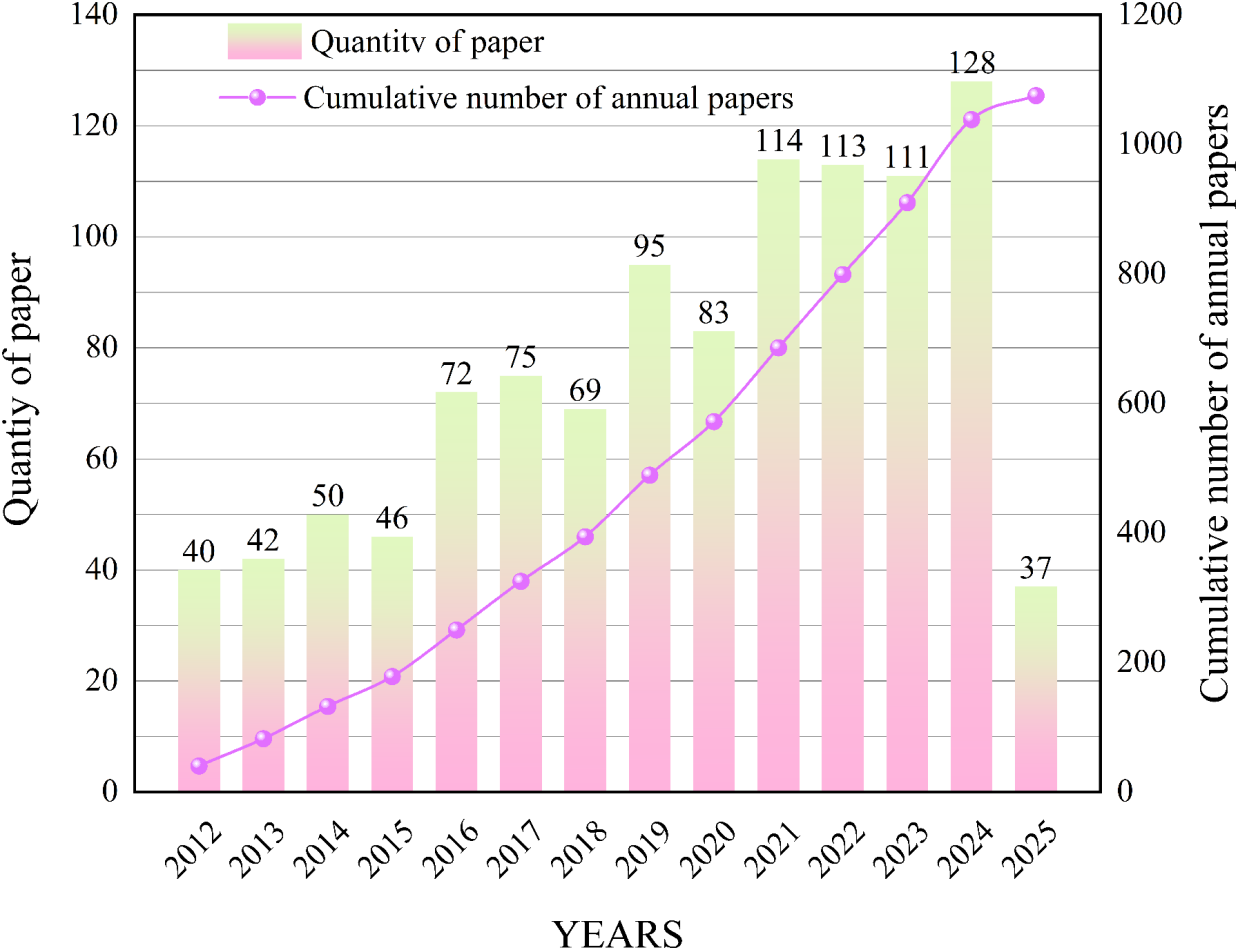
Number of publications in journals and conferences, 2012-2025 (As of October 2025).

Using the above-described methods, literature retrieval and thematic analysis were conducted simultaneously in Web of Science and China National Knowledge Infrastructure (CNKI) to examine research priorities in the field of mission payloads. The results show that with the increasing diversification of UAV mission payloads, related research has exhibited a steady growth trend (**Figure 3**).

## 3 Classification of UAV payloads

Currently, UPT is an essential enabler for extending UAV operational capabilities and enhancing adaptability across diverse scenarios evolving toward greater diversity and functional integration. This chapter systematically classifies and analyzes the structural characteristics and representative application scenarios of two primary payload categories: sensor-based payloads and actuator-based payloads. The aim is to provide a foundational reference and conceptual framework that supports in-depth research and guides future technological development in this field.

### 3.1 Sensor-based payloads

Sensor payloads constitute the essential foundation for UAVs to perform a wide range of operational tasks, playing critical roles in navigation, positioning, environmental perception, and mission execution. Through the integration of multiple sensors and the fusion of their data, UAVs achieve markedly improved situational awareness and operational stability in complex environments. In recent years, related technologies have continued to improve in terms of accuracy, resistance to interference, and integration, providing more reliable technical support for applications in agriculture, emergency response, and other scenarios.

**Figure 4:**
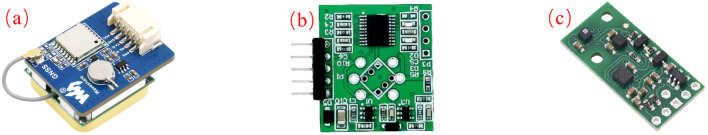
UAV navigation and positioning sensors. **(a)** BDS/GPS positioning system used for global positioning and trajectory tracking, **(b)** MEMS sensors used for attitude estimation and motion detection, **(c)** IMU used for orientation measurement and navigation stabilization.

Among the different types of sensor payloads, navigation and positioning sensors form the basis for stable flight and precise UAV control. Typical examples include inertial sensor inertial measurement unit (IMU), Beidou Navigation Satellite System (BDS)/Global Positioning System (GPS) receivers, and MEMS devices, as shown in **Figure 4**. These sensors provide essential parameters such as attitude, position, and velocity. Studies have demonstrated that combined BDS and GPS positioning achieves better satellite availability, geometric precision, and positioning accuracy compared with single-system solutions (Hong et al., 2020; Tang et al., 2021). In addition, navigation schemes that integrate low-cost MEMS sensors with barometers have been shown, through theoretical analysis, simulation, and experimental validation, to enable high-precision navigation even in complex environments (Liu and Chen, 2020). At the same time, high-precision magnetometer-enhanced IMU simulators have offered a reliable platform for algorithm development, promoting the application of low-cost sensors in challenging scenarios (Brunner et al., 2015).

However, ensuring accurate synchronization and fusion of data remains a challenge when UAVs carry multiple sensors simultaneously. Low-cost sensors often exhibit unstable performance, especially under large temperature variations or during rapid UAV maneuvers, which limits their applications in areas such as emergency response and precision agriculture. To address these issues, solutions involve not only hardware-level time synchronization mechanisms but also real-time monitoring of sensor status and dynamic adjustments. At the same time, establishing unified interface standards is crucial to facilitate the integration and coordinated operation of different sensors.

**Figure 5:**
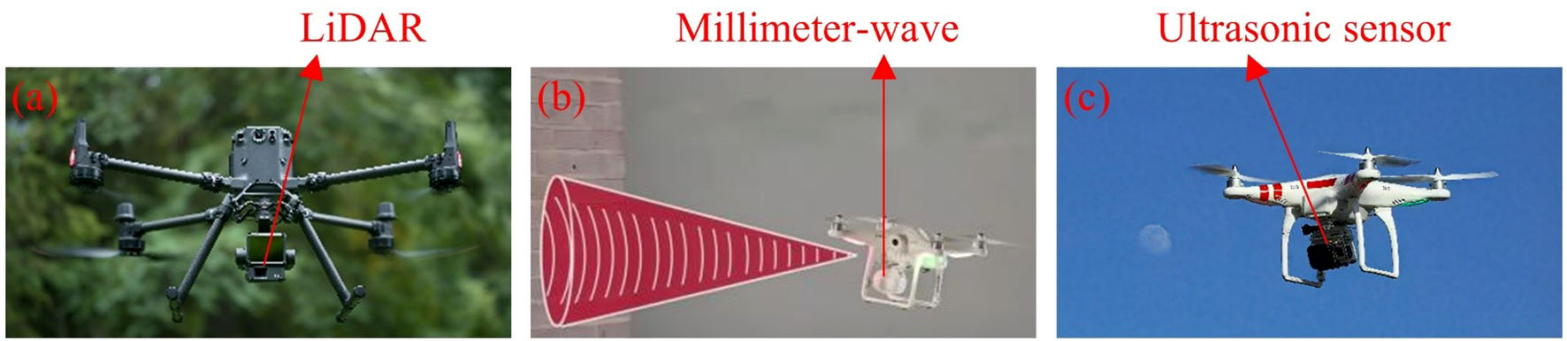
UAV perception sensors. **(a)** UAV equipped with LiDAR for distance measurement, **(b)** UAV equipped with millimeter-wave radar for obstacle avoidance detection, **(c)** UAV equipped with ultrasonic sensors for target tracking.

Environmental perception sensors, which focus on detecting flight conditions and surrounding environments, mainly include light, laser detection and ranging (LiDAR), millimeter-wave radar, and ultrasonic sensors, as illustrated in **Figure 5**. These sensors allow UAVs to perceive physical features of their surroundings, supporting tasks such as obstacle avoidance (Liang et al., 2023; Tang et al., 2023), mapping (Liu et al., 2025), and dynamic target tracking (Xu et al., 2024). For example, Feng et al. (2022) integrated drone-mounted LiDAR with multi-scale cylindrical detection and multi-scale annular fitting techniques, enabling direct extraction of tree trunk points and measurement of diameter at breast height. Analysis of field data showed a trunk extraction accuracy of 95.80% and a DBH measurement R² of 0.708, while simulated data achieved 95.10% accuracy and an R² of 0.882. The study further indicated that scanning angles between 50° and 65°, combined with increased flight path coverage, improved measurement precision. Tung Ng et al. (2023) noted that the use of millimeter-wave radar on UAVs remains at an early stage. Currently, it is often combined with other sensors for obstacle detection and avoidance, which enhances overall performance but also increases payload weight. Research on standalone millimeter-wave radar applications for UAVs is still limited. Obstacle estimation is commonly performed using Kalman filtering, which requires further refinement to address nonlinear challenges. Most obstacle avoidance algorithms rely on geometric methods, which are simple to implement but necessitate further exploration in path optimization and three-dimensional avoidance strategies. Wu et al. (2018) proposed an obstacle avoidance system for a UAV that integrates ultrasonic radar, infrared detection, and visual recognition. The system allows real-time detection of obstacles, such as transmission towers and power lines, during inspections, automatically generating alerts and planning avoidance routes. As a result, it reduces the risk of collisions and operational errors while enhancing the safety and reliability of inspection tasks.

**Figure 6:**
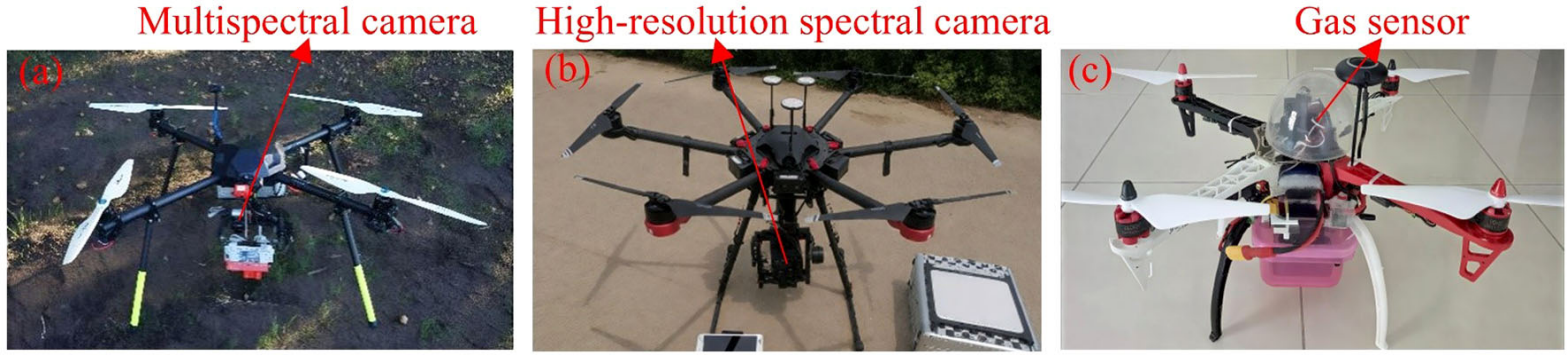
Mission-executing sensor types. **(a)** Multispectral camera mounted on a UAV (Gallego et al., 2018), **(b)** Hyperspectral camera mounted on a UAV (Liu et al., 2021), **(c)** Gas sensor mounted on a UAV (Handayani and Suparta, 2022).

Task-oriented sensors are primarily designed to support the execution of operational tasks and precise control. Common examples include multispectral and hyperspectral cameras, as well as gas detection sensors (**Figure 6**), which play a key role in enabling UAVs to carry out refined operations. The performance of these sensors largely determines the accuracy and applicability of UAV missions. Multispectral cameras, in particular, have been widely employed in agricultural production and monitoring in recent years. For example, they have been used to classify irrigation methods in hybrid maize varieties (de Oliveira et al., 2025) and to monitor land cover types and vegetation conditions (Reinprecht and Kieffer, 2025). Hyperspectral imaging further enhances data dimensionality and interpretability, with studies demonstrating its use in mapping wetland vegetation communities (Du et al., 2021) and identifying hazardous trees in urban environments (Mat Isa et al., 2025). Additionally, to address environmental safety concerns, Souza et al. (2024) integrated a methane sensor onto a UAV platform, enabling rapid detection and monitoring of large-scale gas leaks and improving environmental risk warning capabilities.

In summary, **Table 1** presents the typical applications of UAV navigation & positioning, environmental perception, and task execution payloads, providing technological support for efficient perception and precise operations in complex environments.

**Table 1:** Classification of UAV sensor payloads.

Category	Typical sensors	Weight range	Power requirement	Cost range	Typical applications	References
Navigation & positioning	IMU, GNNS/GPS, MEMS	10 g-300 g	0.5–5 W	$50-$5,000	Attitude estimation, positioning, and trajectory control	(Hong et al., 2020) (Liu and Chen, 2020)
Environmental perception	LiDAR, millimeter-wave radar, and ultrasonic sensors	50 g-2.5 kg	1–60 W	$1,000-$40,000	Obstacle avoidance, surveying, and target detection	(Liu et al., 2025) (Xu et al., 2024)
Task execution	Multispectral/hyperspectral cameras and gas detection sensors	100 g-3.5 kg	3–80 W	$1,000-$20,000	Precision agriculture monitoring, environmental monitoring, and operational accuracy control	(Reinprecht and Kieffer, 2025) (Du et al., 2021)

Weight, power, and cost are estimated values and may vary depending on specific references or actual equipment.

Although integrating multiple types of sensors has expanded UAV operational capabilities, challenges remain in complex environments, including high payload weight, limited space, and restricted endurance. In addition, issues such as data coordination and mutual interference among sensors can compromise operational stability. Future research may focus on improving sensor reliability under high-temperature, high-humidity, or obstructed conditions, enhancing the efficiency of data synchronization and fusion, and optimizing the integrated design of payloads and UAV platforms. These efforts aim to increase the practicality and endurance of UAV systems for long-duration operations, such as agricultural monitoring.

### 3.2 Actuator-type payloads

Driven by the ongoing advancement of UAV technology, the scope of their applications continues to expand. As mission requirements become more diverse and operating environments more complex, UAV-mounted actuators have emerged as a crucial category of payloads for achieving functional breakthroughs. These actuators typically integrate mechanical structures, electronic control systems, and intelligent algorithms (Klausen et al., 2020; Idrissi et al., 2021; Xiaoyu et al., 2023), thereby enhancing both environmental adaptability and operational performance. Based on their functional orientation, they can be broadly classified into three categories: First, the action execution module, which enables precise operations, is used to achieve accurate control and platform motion execution during the UAV’s flight process (Zhao et al., 2025); second, the payload interaction device, used for remote operations and material transport, focuses on the direct physical interaction between the UAV and task objects, such as grasping, releasing, and hoisting (Yu et al., 2021); third, the biomimetic mechanism, which imitates biological features and possesses structural adaptability, can enhance maneuverability in complex terrains (Guo et al., 2024b). With continued technological progress, such actuators have already demonstrated strong application potential in fields such as precision agriculture (Guo et al., 2022a), electrical circuit inspection (Yang et al., 2020), and logistics transport (Zhang et al., 2024).

**Figure 7:**
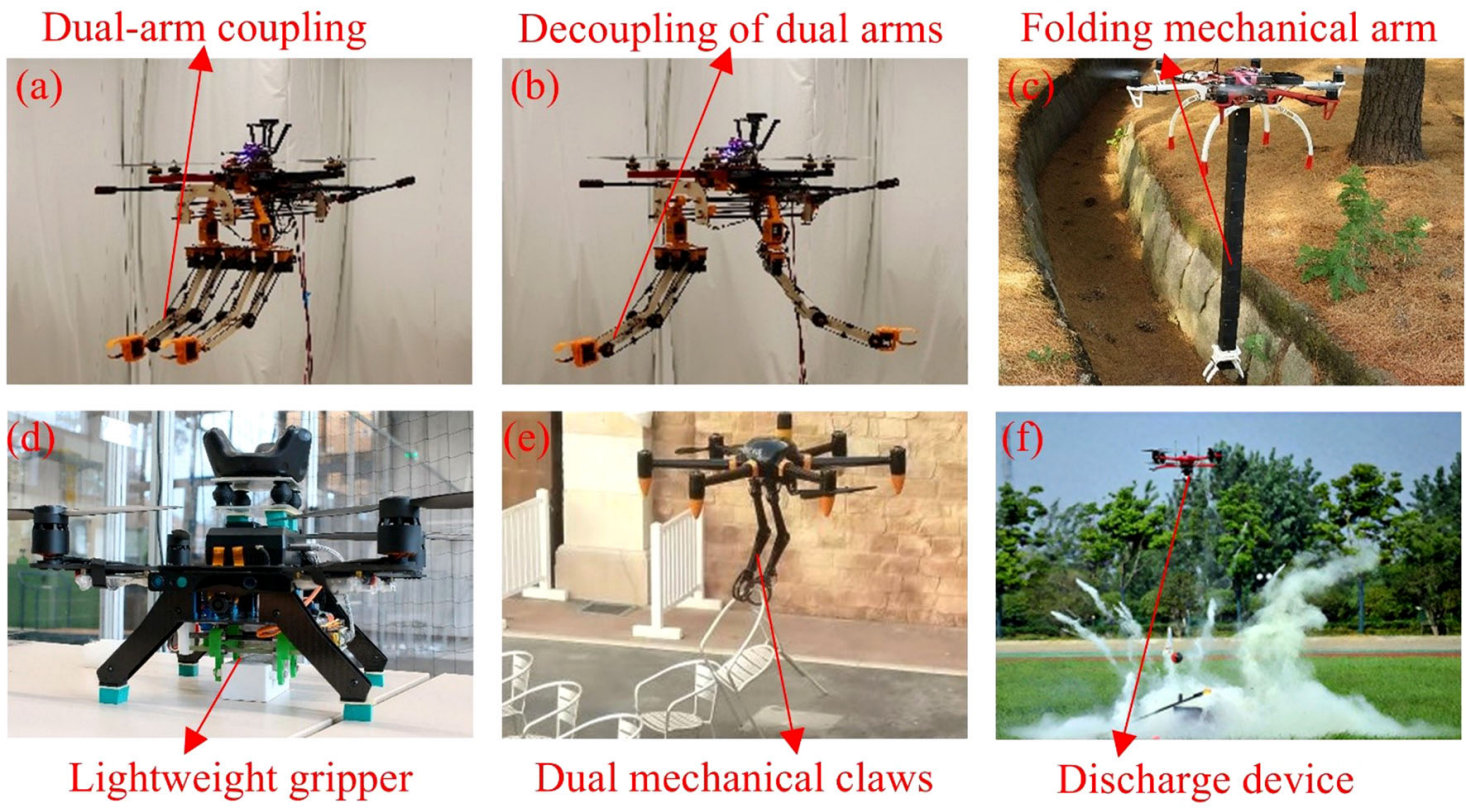
Action execution modules mounted on a UAV. **(a)** Coupled state of dual robotic arms mounted on a UAV (Imanberdiyev et al., 2022), **(b)** Uncoupled state of dual robotic arms mounted on a UAV, **(c)** A foldable robotic arm mounted on a UAV. **(d)** Lightweight gripper mounted on a UAV (Lieret et al., 2020). **(e)** Dual robotic claws mounted on a UAV, **(f)** UAV payload delivery gripper.

Among these, the action execution module serves as a crucial foundation for enhancing the intelligence of drone operations. Typical examples include multi-degree-of-freedom robotic arms (e.g., the dual robotic arms shown in **Figures 7a, b**, and the folding robotic arm shown in **Figure 7c**) (Sarkisov et al., 2019), end-effector grippers (e.g., the lightweight gripper shown in **Figure 7d**, the dual mechanical gripper shown in **Figure 7e**, and the delivery gripper shown in **Figure 7f**) (Chermprayong et al., 2019; HaiBin et al., 2024), and various deployment mechanisms (Buzzatto and Liarokapis, 2024). Robotic arms typically support control modes such as teleoperation, force control, and visual servoing, and can be flexibly switched according to task requirements. Teleoperation is suitable for remote tasks in complex environments, force control meets the needs of high-precision grasping and assembly, and visual servoing facilitates target recognition and autonomous operations in dynamic environments. Additionally, robotic arms can be equipped with various end-effectors to adapt to diverse operational scenarios. As critical end-effectors, grippers often integrate servo motors, electric motors, or pneumatic actuators with force or vision sensors to achieve stable grasping of various targets. Deployment mechanisms are primarily used for material delivery or emergency rescue operations, enabling precise releases through flight control systems or remote operation, thereby enhancing both operational efficiency and safety.

Although action execution modules significantly expand the mission capabilities of UAVs, their design still requires a trade-off between weight and energy consumption. Excessive emphasis on lightweight construction may compromise structural strength and operational precision, whereas excessive functional integration can lead to high power consumption, reducing flight endurance and mission flexibility (Zhang and Kovacs, 2012). To address this, future designs should focus on enhancing the structural integration and task adaptability of execution modules. One feasible approach is to integrate multiple end-effectors within the robotic arm structure to enable rapid switching between tasks, while simultaneously incorporating multi-source perception signals, such as visual and force feedback, to improve target recognition, decision-making, and operational response. This would enhance the stability and practical utility of action execution modules in complex scenarios.

**Figure 8:**
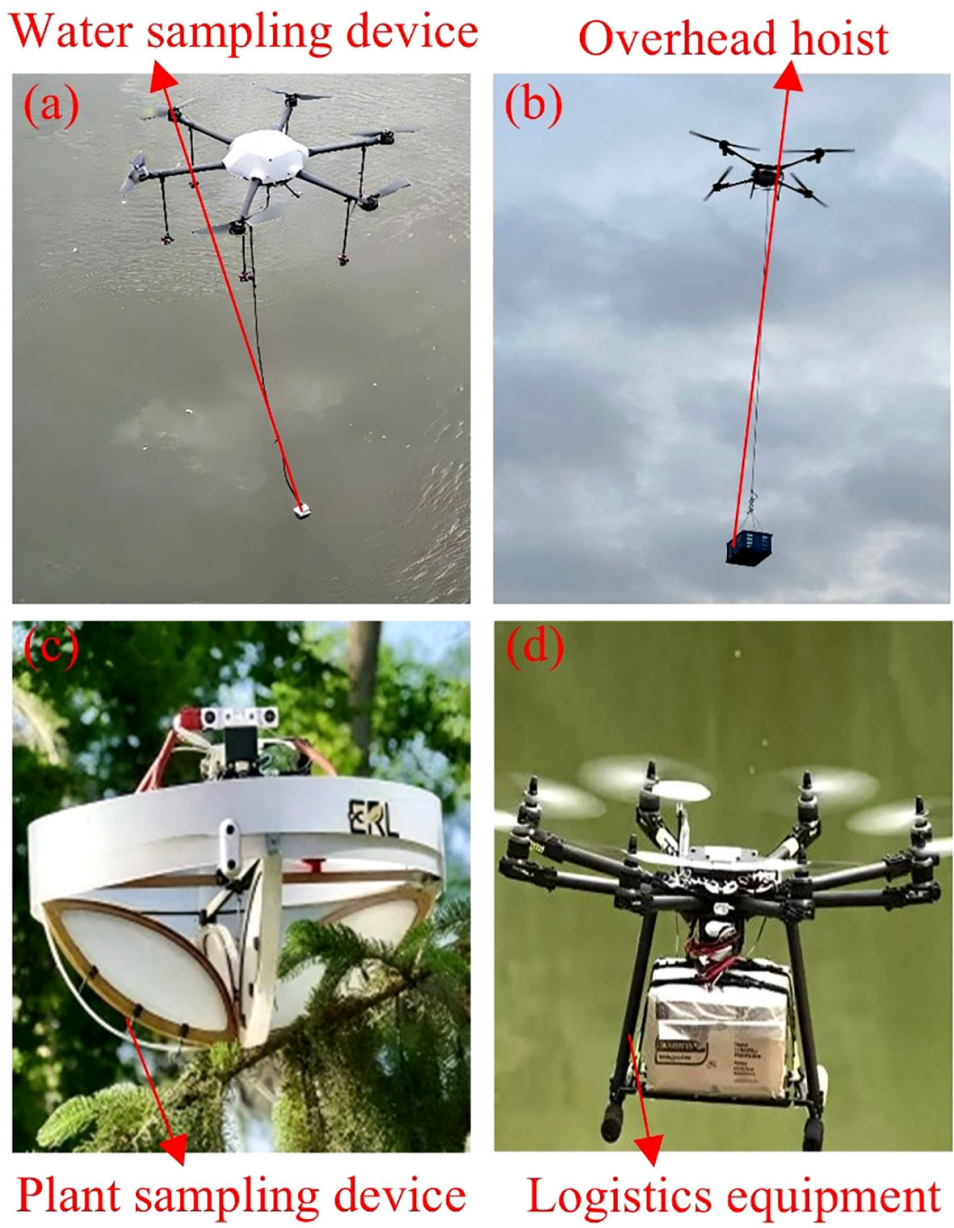
UAV-mounted payload interaction devices. **(a)** UAV equipped with a water sampling device, **(b)** UAV equipped with a hoisting device, **(c)** UAV equipped with a plant sampling device, **(d)** UAV equipped with a logistics transport device.

Payload interaction devices and bio-inspired adaptive mechanisms mounted on UAVs play a critical role in task execution and physical interaction with the environment. They are widely applied in scenarios such as logistics distribution (Liu et al., 2024a), cargo transportation (Sun and Antonio, 2023; Hong et al., 2025), and sample collection (Liu et al., 2023). The typical systems include the water sampling device in **Figure 8a**, the hoisting device in **Figure 8b**, the plant sampling device in **Figure 8c**, and the logistics transport device in **Figure 8d**. Sampling devices mainly cover water samplers (Graham et al., 2022), air samplers (Yang et al., 2023), soil collectors (Klopfenstein and Lussier Desbiens, 2024), and vegetation grippers (La Vigne et al., 2022). These systems relied on intelligent control frameworks to achieve high-precision sampling and environmental monitoring tasks. Lifting devices generally adopt miniature motor-driven reels combined with high-strength steel cables or ropes for suspending goods. They are usually integrated into release ports and can be operated through the flight control system, remote control, or preset programs to enable rapid electro-mechanical release (Xiao et al., 2025). Bio-inspired claw devices draw on the structural principles of organisms such as octopus tentacles (Wang et al., 2025) and avian talons (Zhao et al., 2023), enlarging the contact surface to achieve stable grasping of complex or irregular objects.

In summary, **Table 2** presents the typical specifications and performance of UAV actuation payloads, including motion execution modules, payload interaction devices, and bio-inspired adaptive mechanisms, providing technical support for stable grasping, material transport, and precise operations in complex environments.

**Table 2:** Classification of UAV actuation payloads.

Category	Typical sensors	Weight range	Power requirement	Cost range	Typical applications	References
Motion execution module	Robotic arms (dual-arm/folding), grippers, and delivery devices	0.2–5 kg	5–200 W	$500-$5,000	Precision manipulation, grasping, and deployment	(Zhao et al., 2025) (Sarkisov et al., 2019)
Payload interaction device	Water/soil/vegetation samplers, hoisting devices, and logistics transport modules	0.3–6 kg	5–150 W	$300-$40,000	Sample collection, cargo hoisting, and delivery	(Liu et al., 2024a) (Liu et al., 2023)
Bio-inspired adaptive mechanism	Bio-inspired grippers (octopus tentacles, bird talons)	0.5–3 kg	10–60 W	$500-$3500	Grasping complex or irregular objects	(Wang et al., 2025) (Zhao et al., 2023)

Weight, power, and cost are estimated values and may vary depending on specific references or actual equipment.

Although grippers, sampling devices, and cargo hoisting mechanisms have been preliminarily applied in scenarios such as agricultural harvesting, water sampling, and material transport, several limitations remain, including unstable grasping, inaccurate recognition, and limited adaptability. Grippers are prone to slippage or failure when handling objects of varying shapes and materials, suggesting that flexible materials and biomimetic designs could improve stability. Sampling devices often exhibit positioning errors in complex environments, which may be mitigated by integrating visual and LiDAR sensors to enhance operational precision. Cargo hoisting mechanisms are constrained by payload capacity and swing control, requiring optimization through lightweight structural design and swing suppression algorithms to ensure the stable and safe operation of payloads in challenging conditions. Future research places greater emphasis on the coordinated advancement of structural design and control strategies, intending to ensure the stable and safe operation of mission payloads under complex environmental conditions.

## 4 Applications in agricultural scenarios

**Figure 9:**
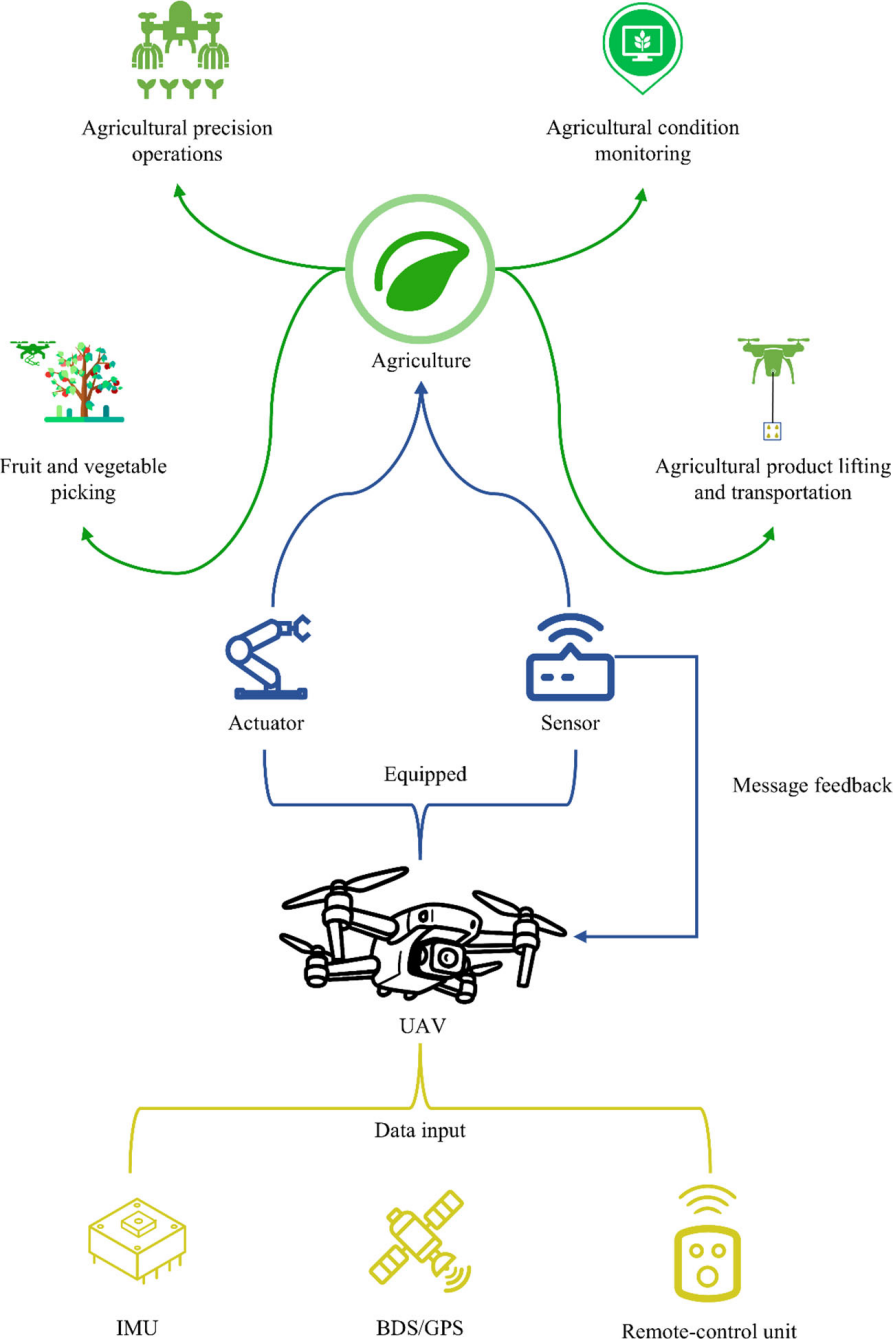
Application scenarios of UPT in agriculture (green), UAV-mounted devices and feedback (blue), UAV data sources (yellow).

The application of UPT in agriculture continues to expand and has become a key means of promoting agricultural modernization and intelligent management. UAVs receive data signals through IMU, BDS/GPS positioning systems, and a Remote-control unit, and execute various agricultural tasks using onboard actuation and sensor payloads. At the same time, the feedback capabilities of multiple sensors enable UAVs to perform operations with greater precision, including crop monitoring (Li et al., 2024a), precision operations (Chen et al., 2025), and the transport or harvesting of agricultural products (Kumar and Behera, 2024), as shown in **Figure 9**. Here, “precision operations” refer to controllable agricultural tasks such as spraying, fertilization, or sow, focusing on operational accuracy and efficiency, whereas “crop monitoring” emphasizes the collection and analysis of data on crop growth, pest conditions, and field environment to support informed decision-making These technologies not only improve the efficiency of precision agricultural tasks but also reduce labor input and operational risks, significantly enhancing the refined management of agricultural production and demonstrating strong application potential and development prospects.

### 4.1 Agricultural condition monitoring

**Figure 10:**
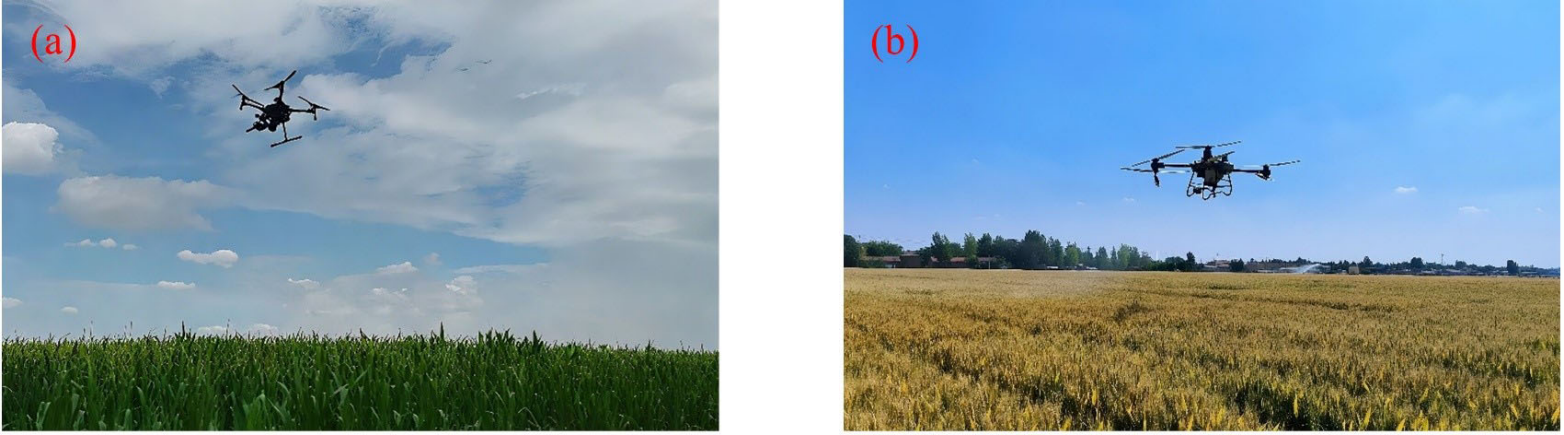
Applications of UAVs in agricultural monitoring. **(a)** Monitoring maize growth using a UAV equipped with multispectral sensors, **(b)** Monitoring rice growth using a UAV equipped with multispectral sensors.

The growth of crops is a highly complex process, and timely as well as accurate monitoring of crop conditions remains essential for maintaining healthy development. In recent years, UPT has found broad application across different regions, crop species, production scenarios, and monitoring indicators (He et al., 2025). Previous studies demonstrated that UAV-based approaches provided clear advantages in crop stress detection (Li et al., 2020b), growth assessment (Gokool et al., 2023), pest and disease identification (Li et al., 2024b; Lu et al., 2024b), and yield estimation (Kim et al., 2024). With these capabilities, farmers are able to gain near real-time insights into crop growth dynamics and emerging problems, as illustrated in **Figure 10**.

In practical crop monitoring, UPT shows wide-ranging application potential. With multispectral, hyperspectral, and thermal imaging sensors onboard, UAVs provide detailed information on crop stress, supporting nutrient assessment and precision field management. For instance, Yu et al. (2023) obtained canopy spectral data of rice using UAV-based hyperspectral sensing and, by combining spectral transformations with machine learning techniques, successfully estimated the rice nitrogen nutrition index, offering valuable data for nutrient diagnosis and fertilizer management. Likewise, Olson et al. (2022) applied UAV-mounted hyperspectral sensors together with regression models to predict nitrogen efficiency indicators, such as whole-plant nitrogen concentration and nitrogen use efficiency in mature maize, thereby extending the scope of UAV applications in crop nitrogen monitoring. Beyond nutrient detection, UAVs equipped with multi-source sensors capture critical information on canopy structure, spectral traits, and texture parameters, enabling real-time crop growth assessment and yield estimation. This approach has been employed in studies of multiple crops, including rice (Luu et al., 2025), maize (Tirado et al., 2020), cotton (Feng et al., 2020), and potato (Bian et al., 2023). Overall, UAV mission payload applications in crop monitoring cover a variety of research directions and practical domains, as summarized in **Table 3**.

**Table 3:** Main research types and applications of UPT in agricultural monitoring.

Research type	Representative sensor/method	Typical crops	Primary research focus and applications	Reference
Crop stress and nutrient monitoring	Multispectral, hyperspectral, thermal imaging	Rice, corn, etc.	Canopy spectral data acquisition, nitrogen nutrition index estimation, and crop nutrition diagnosis	(Yu et al., 2023) (Olson et al., 2022) (Li et al., 2025a)
Crop growth monitoring	Multi-source sensors (multispectral, texture features, etc.)	Rice, corn, cotton, potatoes	Canopy structure and texture parameter analysis, growth condition assessment, and yield estimation	(Iagăru et al., 2022) (Oré et al., 2020) (Ruwanpathirana et al., 2024)
Pest and disease identification	RGB, multispectral, thermal infrared; deep learning models	Wheat, winter wheat, grapes, coffee	Early detection of pests and diseases, multi-source data fusion, and deep learning-based automatic identification	(Su et al., 2021) (Bhandari et al., 2020) (Ye et al., 2025)
Production estimate	UAV imagery + machine learning, regression models	Corn, rice, sugarcane, potatoes	Optimize fertilizer management plans by integrating growth monitoring data with model-predicted yields.	(Li et al., 2025b) (Adeluyi et al., 2022) (Guo et al., 2022b)
Multi-source data fusion	Satellite + UAV + Ground Monitoring	Multiple crops	Constructing a sky-ground integrated monitoring system to enhance spatiotemporal resolution and data accuracy	(Zhou et al., 2021) (Sun et al., 2024)

In summary, **Table 3** outlines the principal research directions and application pathways of UPT in crop monitoring, covering areas such as stress detection, growth assessment, pest and disease recognition, yield estimation, and multi-source data fusion. The integration of multiple sensors with intelligent algorithms enhances both the spatiotemporal resolution and the analytical accuracy of monitoring, thereby offering critical technological support for precision agriculture management.

In an integrated “satellite-UAV-ground” crop monitoring framework, satellites deliver large-scale macro observations, ground sensors provide localized fine-scale measurements, and UAVs, with their flexible flight capability, generate high-resolution data in between. The complementarity of these components ensures broad spatial coverage while retaining fine temporal and spatial resolution, thereby strengthening the practical value of crop monitoring. For instance, Adeluyi et al. (2022) applied UAV-based RGB and multispectral imagery in combination with a random forest model to estimate rice above-ground biomass. In maize production, Guo et al. (2022b) collected vegetation indices, texture indices, and plant height data using UAV-mounted RGB and multispectral cameras and, together with machine learning methods, predicted grain yield and optimized nitrogen, phosphorus, and potassium fertilizer ratios. Oré et al. (2020) employed a UAV-based differential interferometric radar system to monitor maize and sugarcane growth processes and to construct crop growth models. For high-value vegetables, Lee et al. (2023) integrated UAV RGB and multispectral imagery with deep learning algorithms to monitor individual broccoli plants and support precision field management. Similarly, Ruwanpathirana et al. (2024) and Li et al. (2020a) developed growth monitoring and yield estimation models for sugarcane and potato, respectively, using UAV-derived RGB and hyperspectral data, further extending the scope of UAV applications across crop types. In addition, Iagăru et al. (2022) utilized UAV multispectral imagery for integrated monitoring of crop growth, operational quality, and ecological disturbances, providing useful references for sustainable agricultural management.

In summary, UPT is already well established in applications such as crop stress detection, growth monitoring, and yield estimation, and it now serves as a major tool for data collection in crop monitoring. This type of payload, which integrates sensing and intelligent processing, has reshaped traditional approaches to obtaining crop information and provides a foundational support for building digital agricultural management systems. Within the “satellite-UAV-ground” integrated monitoring framework, UAVs serve as intermediate-scale platforms connecting satellite and ground observations. Leveraging high-resolution sensing and flexible operational capabilities, they effectively bridge the gap between large-scale and fine-scale observations, improving both the efficiency and accuracy of crop data acquisition. However, challenges remain under complex weather conditions, extensive field environments, and long-duration operations, including unstable data quality and limited system endurance. Future research should focus on further enhancing multi-sensor integration and platform intelligence, expanding adaptability across different crops and cultivation patterns, and promoting the transition of UAV technology from a standalone monitoring tool to a component of intelligent agricultural decision-support systems.

Beyond growth monitoring and yield estimation, UPT also demonstrates significant advantages in pest and disease detection. UAV-based pest and disease identification involves various technologies and methods, with the main sensor types and applications summarized in **Table 4**.

**Table 4:** Summary of key technologies and applications for UAV-based pest and disease detection.

Technology category	Sensor type	Typical crops	Main methods and applications
Optical image recognition (Su et al., 2021)	RGB and multispectral	Wheat, Grape, Coffee	Image-based pest and disease detection; Deep learning classification
Thermal infrared monitoring (Zhang et al., 2025)	Thermal imaging sensors	Various crops	Early stress and disease detection
Multi-source data fusion (Zhou et al., 2021)	Satellite + UAV + Ground-Based monitoring	Multiple crops	Multi-scale information fusion to enhance recognition accuracy
Deep learning models (Li et al., 2023a)	CNN, Transformer, etc.	Various crops	Automatic feature extraction and classification for Real-time recognition

Traditional reliance on manual field inspection is not only inefficient but also struggles to capture early signs of infestations. In contrast, UAVs offer high mobility and broad coverage, and when equipped with visible, multispectral, and thermal infrared sensors, they can efficiently capture anomalous changes in crops, providing effective support for early detection and precise control of pests and diseases. For example, Su et al. (2021) used a RedEdge multispectral camera combined with a U-Net deep learning network to accurately identify wheat stripe rust, demonstrating the effectiveness of integrating multispectral imaging with semantic segmentation for field monitoring. Similarly, Bhandari et al. (2020) employed high-resolution RGB imagery along with vegetation indices such as NDVI and green indices to quantitatively assess leaf rust severity in winter wheat, highlighting the practical applicability of visible-light sensors.

Building on this foundation, several studies investigate multi-source data fusion as a way to improve spatial accuracy and regional adaptability in pest and disease detection. For instance, Zhou et al. (2021) combined UAV-acquired Parrot Sequoia + multispectral imagery with Sentinel-2 vegetation index data and applied a support vector regression model to analyze vineyard disease incidence, thereby extending the role of UAVs in orchard monitoring. Integrating data from different remote sensing platforms enhances the precision of image analysis and helps overcome the coverage and revisit limitations of single systems, thus improving the adaptability of UAV-based monitoring.

Beyond multi-source data fusion, recent research increasingly emphasizes the use of advanced algorithms to enable more intelligent analysis of fused datasets. With the development of deep learning, UPT has evolved from basic feature extraction to automatic classification in pest and disease detection. For example, Li et al. (2024b) developed a convolutional neural network model that achieved high-precision classification of crop pests and diseases. Building on this work, Li et al. (2023a) introduced the MCD-Yolov5 model, which integrates multi-layer feature fusion, the convolutional block attention module, and the detection Transformer, markedly improving both the speed and robustness of detection. To address the diverse challenges posed by different crop types and dynamic field conditions, ongoing studies continue to refine the specificity and adaptability of recognition models. For instance, Maruthai et al. (2025) applied a hybrid visual graph neural network for early detection of coffee pests, including coffee berry borers and mealybugs, leveraging both image features and the structural relationships among pests.

The above studies indicate that UPT, when equipped with multiple sensor types, has been widely applied in crop pest and disease detection, enhancing monitoring efficiency and opening new avenues for precision management. Nevertheless, challenges persist in situations involving multiple simultaneous diseases, similar symptom expression, or complex field conditions, where identification accuracy may be limited. Environmental factors such as variations in illumination and vegetation interference can further affect image quality, constraining large-scale deployment. Future research should prioritize improving multi-sensor integration and developing cooperative multi-UAV detection strategies to enhance system stability and adaptability under complex conditions.

### 4.2 Precision agricultural applications

With the widespread adoption of UPT in recent years. It has shown significant improvements in operational efficiency and quality for agricultural tasks such as sowing, pesticide application, and fertilization. Its high efficiency and flexible operation modes help address traditional agricultural challenges, including low efficiency, limited precision, and high labor dependence. Building on this, recent studies examine the specific applications of UAV mission payloads in precision agriculture, as well as innovations and enhancements in related technologies and devices. By integrating seeding units, spraying systems, and intelligent control modules, UAVs have efficiently carried out precision operations such as sowing (Wu et al., 2020), pesticide application (Desen Köycü et al., 2024; Yu et al., 2025), and fertilization (Yu et al., 2022), particularly in complex terrains that are difficult for conventional machinery to access. For an overview of UAV mission payload applications in precision agriculture, **Table 5** provides a concise summary of the main application types and key technologies.

**Table 5:** Main application types and key technologies of UPT in precision agriculture.

Application types	Key technologies and devices	Main operations	Reference
Seeding	Seeder structure improvement, path optimization, and intelligent control	Seeder structure improvement, path optimization, and intelligent control	(Dampage et al., 2020) (Maldonado et al., 2025)
Pesticide application	Spraying system integration, spray parameter optimization, and intelligent monitoring	Precision pesticide application and pest/disease control	(Guo et al., 2021) (Guo et al., 2024a) (Yallappa et al., 2024)
Granular fertilizer application	Precision fertilization and pest/disease control	High-precision fertilization and efficient nutrient utilization	(Su et al., 2022) (Zhou et al., 2024)

**Figure 11:**
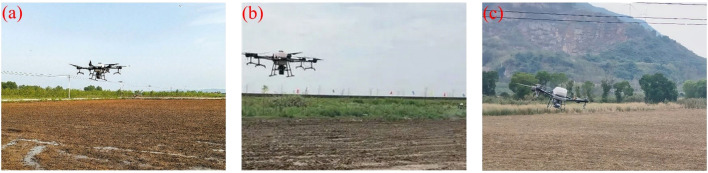
Applications of UAV in seeding. **(a)** Seawater rice seeding, **(b)** Rice seeding, **(c)** Wheat capsule seeding.

In sowing applications (illustrated in **Figure 11a** for saline rice seeding, **Figure 11b** for rice transplanting, and **Figure 11c** for wheat hill sowing), research has mainly concentrated on enhancing the design of seeding devices, optimizing operational paths, and achieving precise seed distribution. Md. Abu Jubair et al. (2018) developed an autonomous UAV system for efficient automatic sowing along pre-defined flight paths, achieving a sevenfold improvement in efficiency compared with conventional manual sowing. Dampage et al. (2020) developed a UAV equipped with a dedicated seeder and shutter control technology, enabling automatic or manual seeding in soft-soil paddy fields. The system ensured accurate distribution of 2–3 seeds per drop, effectively meeting rice field planting standards. In terms of path optimization, Lu et al. (2023) proposed a cloud-based precision mapping system for unmanned rapeseed seeding, which significantly improved field coverage and reduced missing-seeding rates, providing a technical reference for small-and medium-sized rapeseed fields in southern China. More recently, Maldonado et al. (2025) introduced a modular seeding control system assisted by geospatial data, integrating PWM-based rate control. Their approach reduced seed usage by approximately 40% while enhancing vegetation uniformity and resource efficiency.

Despite the promising prospects of UPT in seeding operations, several key challenges remain unresolved. On the one hand, most crops have strict requirements for seeding depth, whereas current UAV-based spreading devices generally adopt a broadcasting approach, making it difficult to accurately place seeds into the soil and thereby compromising seedling uniformity. On the other hand, the operation is easily influenced by meteorological factors such as wind speed and rainfall, resulting in unstable seeding uniformity and germination rates. To overcome these bottlenecks, pelleting technology can be employed to encapsulate seeds into capsule-like structures, enhancing their resistance to environmental disturbances and simulating the effect of manual seedbeds. In addition, integrating UAVs with ground robots to establish an “air-ground collaborative” operation mode enables functions such as precise seeding position localization, lightweight furrow opening, and soil covering. These approaches improve seeding precision and operational reliability, thereby facilitating the wider adoption of UPT in precision agriculture.

**Figure 12:**
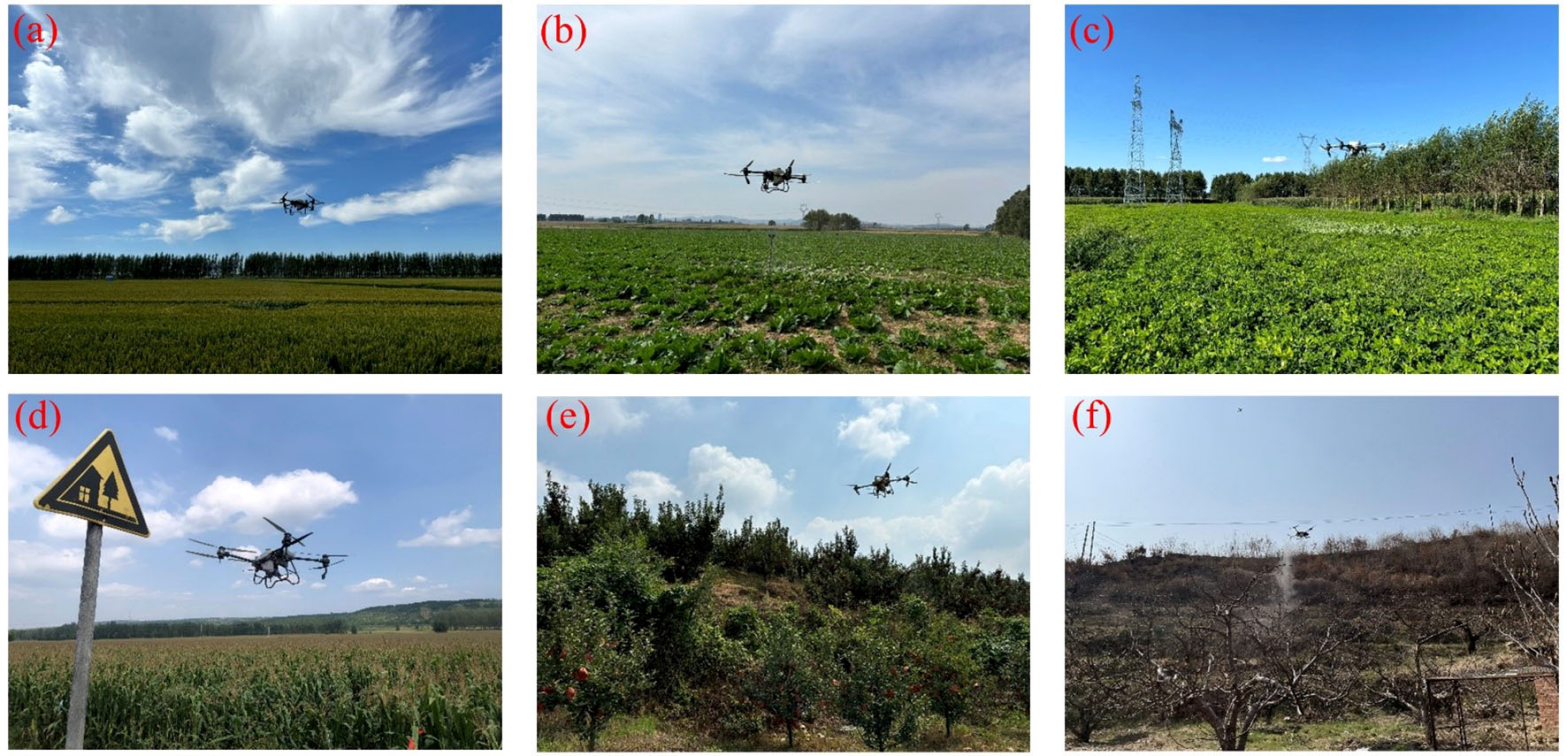
UAV applications in pesticide spraying for different crops. **(a)** Rice, **(b)** Cabbage, **(c)** Peanut, **(d)** Maize, **(e)** Apple orchard, **(f)** Nanguo pear orchard.

Beyond sowing, pesticide application represents another well-established use of UPT. As shown in **Figure 12**, typical crops for UAV-based spraying include rice, maize, peanuts, Chinese cabbage, apples, and Nanguo pears. By integrating spraying systems with precision control modules, this technology improves pesticide utilization and treatment efficiency, making it particularly suitable for areas that are difficult to access with conventional machinery or prone to drift risks.

For example, in the context of pesticide application, Ragiman et al. (2024) applied Different insecticides and fungicides in combination through a UAV platform for rice pest and disease control, and the results were compared with those of conventional sprayers. The findings showed that the UAV-based application offered clear advantages in both effectiveness and efficiency. Likewise, Li et al. (2020c) applied chlorantraniliprole-containing formulations in almond orchards using UAV and conducted a comparative study with ground-based air-assisted sprayers, confirming the advantages of UAV spraying for pest management in high-value crops.

To accommodate different crops and application scenarios, research continues to expand UAV system configurations and spraying strategies. Samseemoung et al. (2024) introduced a locally assembled, low-cost UAV platform equipped with spray arms and a modified RGB camera, which was used to monitor coconut trees for rhinoceros beetle infestations and to carry out targeted pesticide applications. Lopes et al. (2024) applied UAVs repeatedly to distribute Beauveria bassiana conidia in maize and soybean fields to control fall armyworm and soybean looper, and comparisons with backpack sprayers demonstrated the feasibility of UAVs for biological pest management. In addition, Sreenivas et al. (2024) study further indicated that UAV spraying of mixed formulations effectively controlled sap-sucking pests in cotton and maintained excellent control efficacy even with a 25% reduction in dosage, thereby supporting green plant protection strategies.

Moreover, fine-tuning spray parameters remains a crucial strategy for enhancing the precision of UAV-based pesticide applications. Zhang et al. (2020) conducted field experiments with a single-rotor UAV and identified an optimal configuration of 6.0 m flight altitude and 2.5 m/s flight speed for sugarcane spraying, which significantly reduced pesticide use to 15.38 L/ha. Chen et al. (2022) further found through indoor and outdoor measurements that, in close-formation operations of multiple UAVs, synchronous and sequential spraying modes exhibited noticeable differences across field zones, with short-interval cooperative spraying effectively improving droplet deposition uniformity, providing experimental support for UAV swarm operations optimization. Meanwhile, Butler-Ellis et al. (2025) evaluated the drift characteristics of the XAG P40 UAV, showing that ground deposition drift was comparable to that of air-assisted orchard sprayers, while airborne droplets were substantially reduced, making it suitable for areas with strict environmental requirements. Similarly, Sánchez-Fernández et al. (2023) conducted trials in Mediterranean olive orchards and found that UAV spraying systems reduced drift distances by approximately 2.54 times compared with conventional orchard sprayers, effectively lowering buffer zone contamination risk and enhancing environmental friendliness.

Existing studies indicate that UPT offers significant advantages in pesticide application systems by improving operational efficiency, reducing chemical usage, and minimizing environmental impact. However, practical applications still face challenges such as insufficient spray penetration, uneven liquid distribution caused by airflow disturbances, and limitations in payload capacity and flight endurance. To address these issues, future research should explore coordinated operations between UAV and ground equipment and integrate technologies such as electrostatic spraying to enhance spray uniformity and penetration. These strategies are expected to promote wider adoption of plant-protection UAVs in precision agriculture.

**Figure 13:**
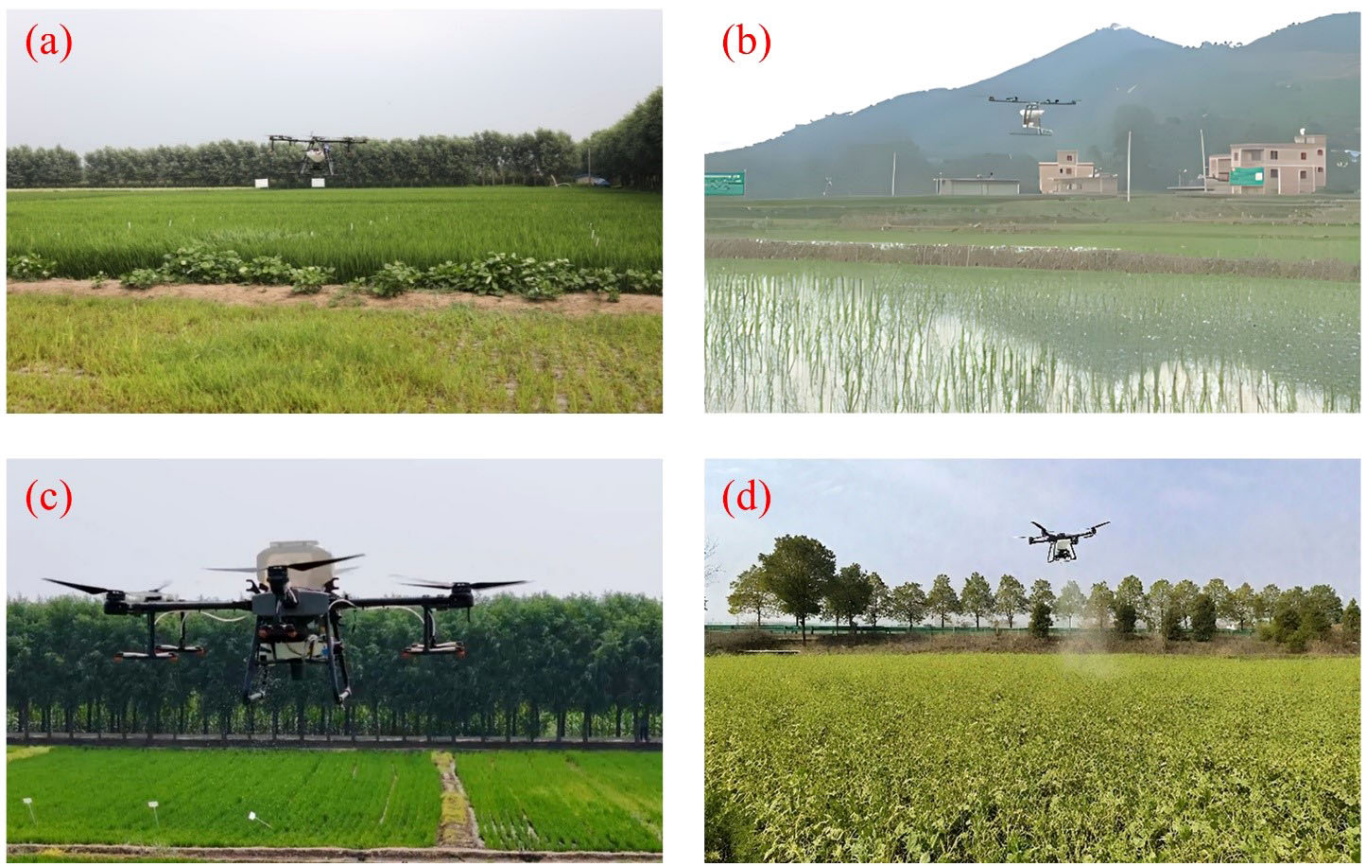
UAV applications in agricultural fertilization. **(a)** UAV for spreading granular fertilizer, **(b)** Particle fertilizer UAV equipped with variable rate control system (Song et al., 2021), **(c)** Variable rate fertilizer UAV, **(d)** UAV spraying of foliar fertilizer on rapeseed leaves.

Beyond pesticide application, UAVs are increasingly being employed for fertilizer application in agriculture, demonstrating notable advantages in precise nutrient delivery and improved fertilizer-use efficiency. As shown in **Figure 13**, UAVs equipped with different spreading systems are applied to typical crops such as rice and rapeseed. Related studies focusing on different crop characteristics and operational conditions-such as rice (Abd. Kharim et al., 2019) and maize (Hernandez-Garcia et al., 2025), have continuously advanced the structural optimization of fertilization devices and the development of intelligent control systems. For example, Zhou et al. (2024) conducted field orthogonal experiments and optimized combinations of deflector opening, disc speed, and flight altitude in a centrifugal disc fertilization system, achieving highly uniform and precise fertilization in rice fields. Xunwei et al. (2024) designed a high-capacity rotary disc fertilizer spreader, and through structural improvements and discrete element method (DEM) simulations, effectively enhanced fertilizer distribution uniformity and operational stability. To further enable variable-rate fertilization, Alameen et al. (2019) developed a granular fertilizer application control system that automatically sets the desired application rate within a speed range of 3–12 km/h, maintaining an error margin of ± 2.6% and demonstrating good adaptability.

Meanwhile, the integration of remote sensing data with crop models has emerged as an important direction in precision fertilization research. Jin et al. (2024) combined UAV-acquired RGB image texture features with the RiceGrow model to establish a variable-rate fertilization decision-making system for rice, generating prescription maps to guide fertilization schemes and achieving both efficient nitrogen utilization and stable yield. Su et al. (2022) further proposed a variable-rate fertilization system based on a single-neuron PID controller; under optimized parameters of 2 m flight altitude and 600 r/min disc speed, the relative fertilization error was reduced to 7.02%, and uniformity was significantly improved. In addition, Gao et al. (2024) applied UAV-based foliar zinc spraying, which significantly increased wheat yield and grain zinc content, achieving simultaneous improvements in productivity and quality, and expanding the technological pathway for micronutrient interventions. In terms of spreading device design, Cancan et al. (2018) developed a pneumatic fertilization system for rice and optimized key components through simulation, enhancing system stability and spreading performance. Overall, existing studies have made substantial progress in variable-rate control, device structure, operational precision, and fertilization strategies, providing a solid foundation for the efficient application of UAV fertilization technology across different crop scenarios.

Overall, UPT has reached a relatively mature stage in precision agricultural operations such as seeding, fertilization, and pesticide application. The aforementioned studies have made substantial progress in the design of operational devices, the precision of variable-rate control, and the development of fertilization strategies, thereby improving both operational efficiency and accuracy. However, practical applications still face common challenges, including uneven fertilization, limitations in payload capacity and flight endurance, and droplet drift. Future research could focus on lightweight and precision-controlled device designs to enhance operational uniformity and resistance to environmental disturbances. In addition, integrating remote sensing monitoring with variable-rate prescription maps could enable intelligent decision-making systems for “image-guided” operations. Furthermore, the development of multifunctional, integrated payloads would improve platform versatility and resource utilization, providing strong support for the advancement of smart agriculture.

### 4.3 Crop harvesting and aerial transport

In mountainous orchard management, steep slopes and narrow roads pose significant challenges for the harvesting and transport of mature fruits. Traditional reliance on manual picking and backpack transport not only involves high labor intensity and low efficiency but also increases the risk of fruit bruising, negatively affecting quality and market value. The rapid development of UPT offers a new approach to this problem, and research is gradually shifting from standalone harvesting to integrated harvesting and transport operations.

In the harvesting stage, researchers have proposed various UAV solutions tailored to different crops and environments. Wang et al. (2024) achieved precise recognition of strawberry fruits and flowers using an improved YOLOv8 model, with accuracies exceeding 82%, and optimized harvesting priority assessment through region segmentation and peak detection. Haydar et al. (2024) employed a UAV equipped with high-precision sensors to generate digital elevation models (DEMs) of blueberry plantations, with RMSEs ranging from 0.36 to 1.04 cm, along with plant height maps, providing reliable operational parameters for automated harvesters. In terms of operational performance optimization, Zhou et al. (2025) enhanced UAV lift coefficients by 11% and significantly improved stability through aerodynamic optimization. Omar and Mukras (2024) developed a UAV with a mechanical arm capable of high-precision cutting of date palm branches under safe conditions, reducing the need for manual climbing.

**Figure 14:**
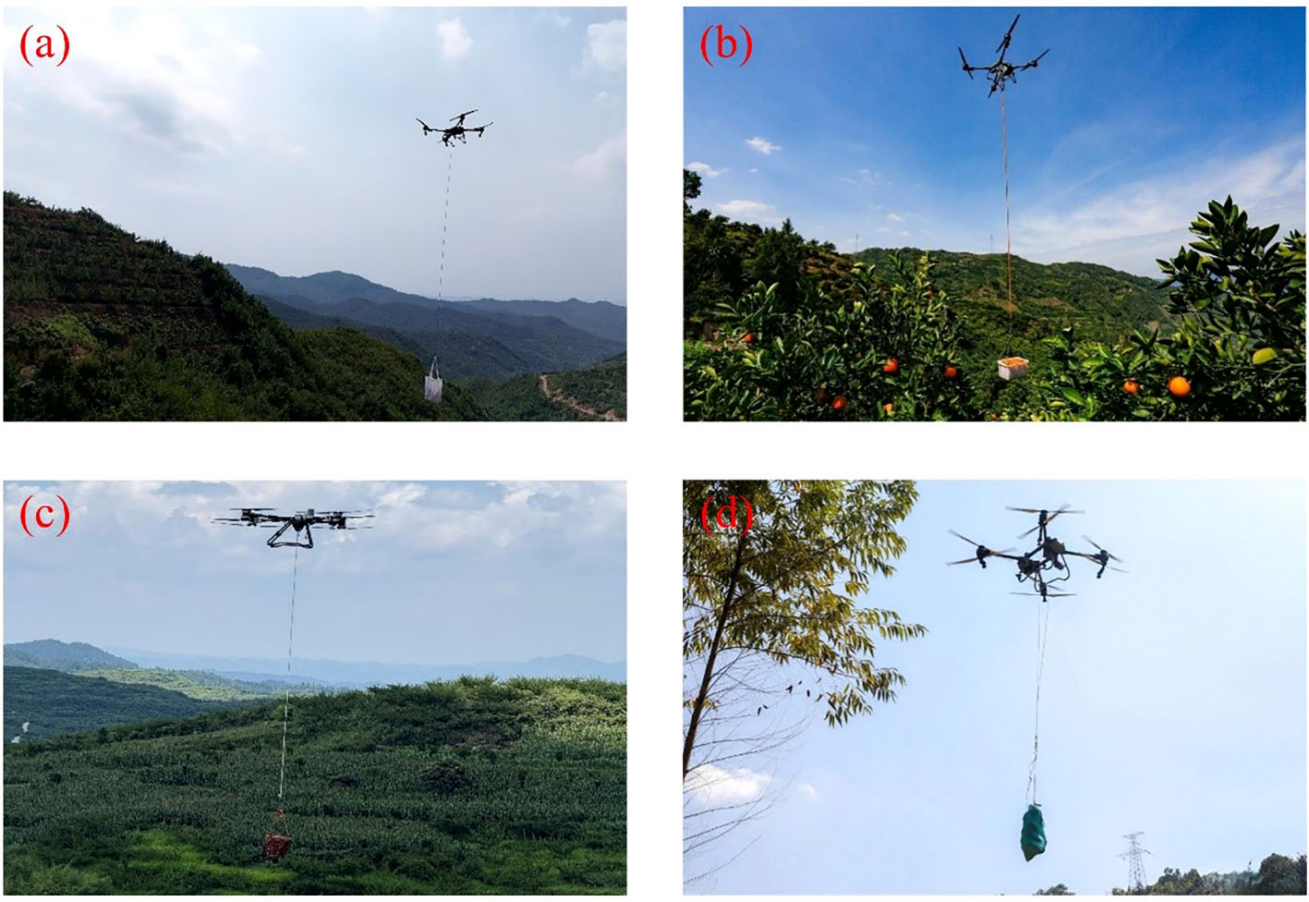
UAV applications in agricultural product transport. **(a)** Nanguo pear, **(b)** Tangerine, **(c)** Peento, **(d)** Pomelos.

As harvesting technologies mature, the industry is increasingly focusing on reducing multiple post-harvest handling steps and achieving direct integration of harvesting and transport. In the hilly regions of Northeast China, fruit trees such as Nanguo pear and apple are distributed across undulating terrain, where post-harvest transport has long relied on manual labor or handcarts. To address this, some orchards have explored UAV-based aerial transport, enabling harvested fruits to be delivered directly to collection points (e.g., aerial transport of Nanguo pears in **Figure 14a**, oranges in **Figure 14b**, flat peaches in **Figure 14c**, and pomelos in **Figure 14d**), thereby reducing intermediate handling and improving overall efficiency.

For example, Duan et al. (2024) developed a UAV-based aerial transport system for banana harvesting in mountainous areas, featuring autonomous grasping and unloading capabilities. Field trials showed an average transport success rate of 83.33% and a transport speed of 0.99 m/s, achieving over three times the efficiency of manual backpack transport without causing fruit damage, demonstrating its potential for efficient, low-loss operations. In industrial practice, several companies have actively advanced UAV-based aerial transport systems. Dajiang Innovation (Shenzhen) launched the T100 agricultural UAV, which can carry a maximum load of 85 kg and is equipped with automatic swing elimination, weight detection, and autonomous flight to loading/unloading points, accommodating diverse agricultural transport needs. The Dajiang Innovation FLYCART100 (DjiFC100) professional aerial transport UAV has a maximum payload of 80 kg and features electric hook operation, wireless charging, and real-time weighing, demonstrating high practicality in agricultural product transport and material delivery (www.dji.com). Additionally, Guangzhou XAG Technology Co., Ltd. developed the XAG Ruiyun 2, also capable of lifting 80 kg, providing further options for transport in small-and medium-sized orchards (www.xa.com).

Although some practical progress has been made, systematic research on UAV-based crop harvesting and aerial transport remains limited. In the harvesting stage, while improvements to YOLO network architectures have achieved high recognition accuracy across various crops, adaptability under strong light, occlusion, and intercropping conditions is still limited, and the end-effectors of robotic arms require enhanced compatibility with different fruit shapes. In the transport stage, although high efficiency and low damage rates have been demonstrated for specific crops, the generalizability and loading/unloading efficiency across different types of fruit crates and varying payloads still need optimization. Future developments could focus on adjustable grasping mechanisms and standardized containers, incorporating magnetic or quick-lock structures to reduce reliance on recognition accuracy. Additionally, integrating LiDAR, stereoscopic vision, and path-optimization algorithms could enhance obstacle avoidance and operational stability in complex environments, achieving efficient integration of harvesting and aerial transport.

## 5 Other typical low-altitude applications

**Figure 15:**
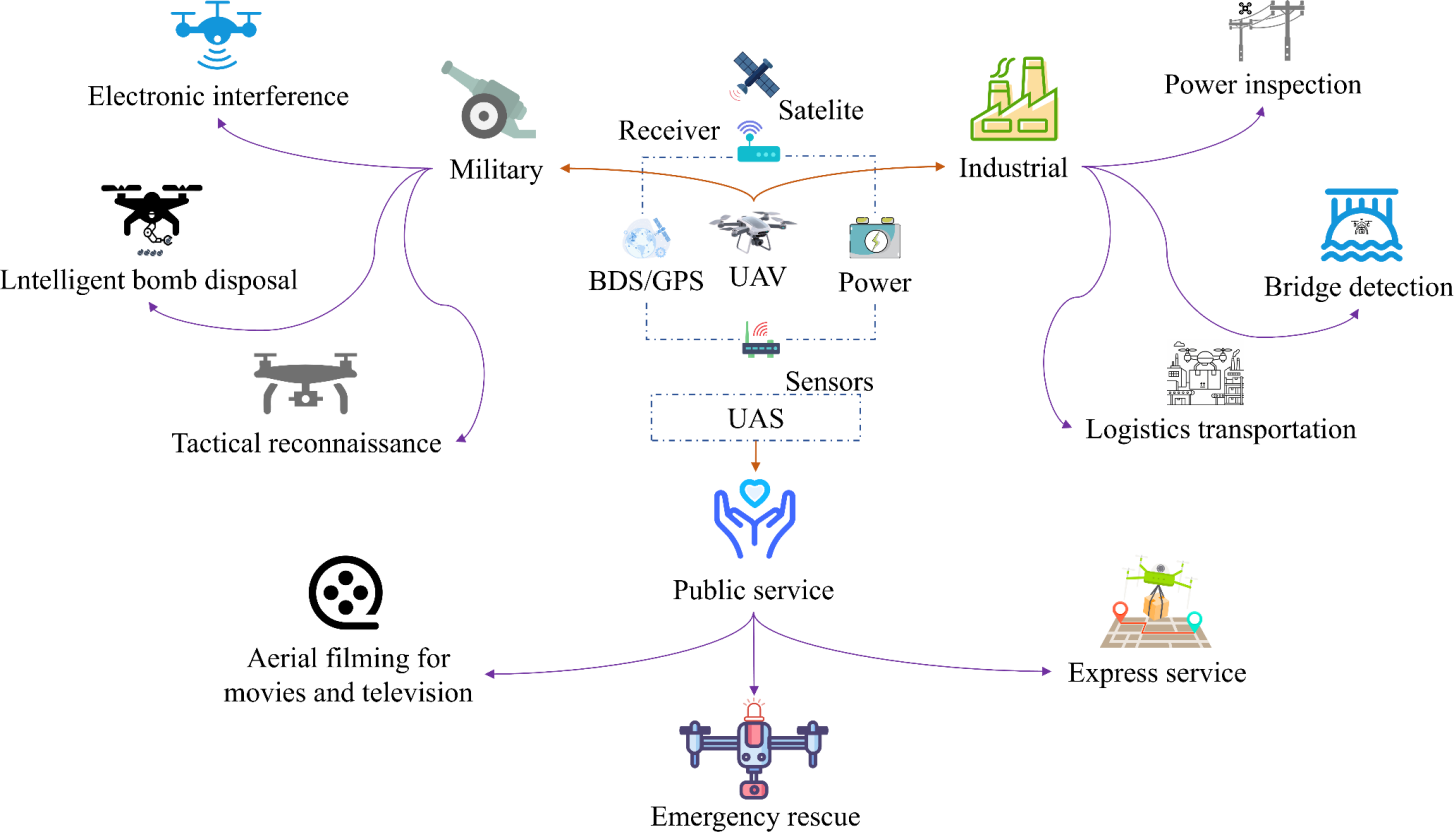
Applications of UPT in other typical low-altitude scenarios.

UPT is not only widely applied in agricultural scenarios but also plays an important role in industrial, military, and public service contexts, as illustrated in **Figure 15**. In the industry, UAVs are primarily used for power line inspection, material transport, and engineering surveying. In military applications, they undertake reconnaissance and target identification tasks. In public services, UAVs are employed for medical supply delivery, emergency rescue, and traffic management. The following sections briefly review the current applications and developmental trends of UPT in these scenarios.

### 5.1 Industry

Currently, traditional industrial sectors are rapidly transitioning toward intelligent operations, placing higher and more diverse performance demands on UPT. As the core module executing specific tasks, UAV payloads largely determine operational capability and application scope. Thanks to their flexible integration and convenient deployment, UAVs have been widely adopted in key industrial applications, including power line inspection (Oladokun et al., 2020), logistics transportation (Zieher et al., 2024), engineering surveying (Jimenez-Cano et al., 2017), and industrial mapping (Pepe et al., 2022; R. et al., 2022), demonstrating strong potential for further development.

#### 5.1.1 Power line inspection

Traditional power line inspection primarily relies on manual visual checks or handheld devices to examine transmission lines and substation equipment section by section. This approach is inefficient, offers limited coverage, and is easily affected by weather and terrain, posing certain safety risks to personnel. Although some regions have introduced helicopters and inspection robots to improve efficiency, these solutions are costly and require high operational and maintenance expenses, limiting their large-scale adoption (Ekren et al., 2023).

**Figure 16:**
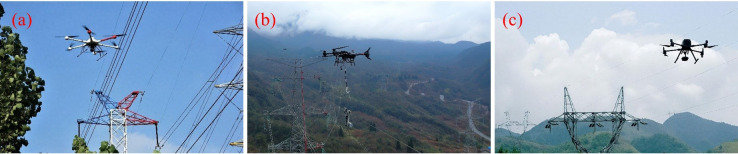
UAV applications in power line inspection. **(a)** Manual remote-controlled inspection, **(b)** UAV with robotic live-line maintenance, **(c)** Remote-controlled programmed automated inspection.

Increasing interest in UPT has driven the gradual automation and intelligent development of power line inspection, offering a novel solution for the field, as illustrated in **Figure 16**. By outfitting UAVs with payloads such as RGB cameras (Wang et al., 2023), infrared thermal imagers (Lee and Park, 2019), and LiDAR sensors (Guan et al., 2021), inspections of power facilities can be conducted efficiently, accurately, and with minimal human intervention. This approach not only enhances operational safety but also markedly improves efficiency while reducing labor and maintenance costs.

In complex operational scenarios, researchers increasingly investigate the contact-based capabilities of UAVs. Jimenez et al. (2020) integrated a lightweight compliant manipulator, POSITRON, into a UAV platform and combined it with an IMU and barometer to achieve precise positioning and attachment on power lines. Torque control was employed to minimize contact disturbances, providing an effective approach for inspection in challenging environments. Gonçalves et al. (2024) proposed combining a semi-autonomous robot with a UAV, where the UAV carries the robot to high-voltage transmission lines for flame-based obstacle clearance. Field tests on 138 kV lines demonstrated strong performance, notably enhancing safety and efficiency. In fully automated inspection systems, Li et al. (2023b) developed an autonomous inspection framework integrating path planning, sliding mode control, and an improved YOLOX model; together with an intelligent docking station, it enables automatic battery replacement and continuous all-weather operation. Takaya et al. (2022) employed a quadrotor UAV with PID control to autonomously track transmission lines and capture images, allowing defect detection and vegetation monitoring, thereby providing a practical solution for automated power facility inspection. In a separate study, Takaya et al. (2024) evaluated fuzzy PID control under strong wind conditions, showing that this method offers superior stability in complex environments and is suitable for extended field inspection tasks.

In summary, UPT has made substantial progress in power line inspection, evolving from basic image acquisition to multifunctional platforms that integrate autonomous flight, intelligent recognition, path planning, and contact-based operations, thereby improving both inspection efficiency and safety. Extensive field tests have validated its effectiveness and supported practical deployment. Despite these advances, UAVs continue to face several challenges in power line inspection. Adverse weather conditions can compromise flight stability and sensor accuracy, while complex terrains in mountainous and forested areas may interfere with communication signals. In addition, limited battery life constrains both the inspection range and operational duration. Future research should focus on enhancing anti-interference communication technologies to ensure stable signal transmission, optimizing flight control systems to improve responsiveness to wind variations and unexpected conditions, and enhancing battery performance or developing rapid battery replacement solutions to extend continuous operation time. Additionally, exploring multi-UAV cooperative inspection with task allocation and coordination could further improve overall efficiency and reliability. These measures can enable UAVs to perform power line inspections more stably and efficiently in complex environments.

#### 5.1.2 Logistics and transport

**Figure 17:**
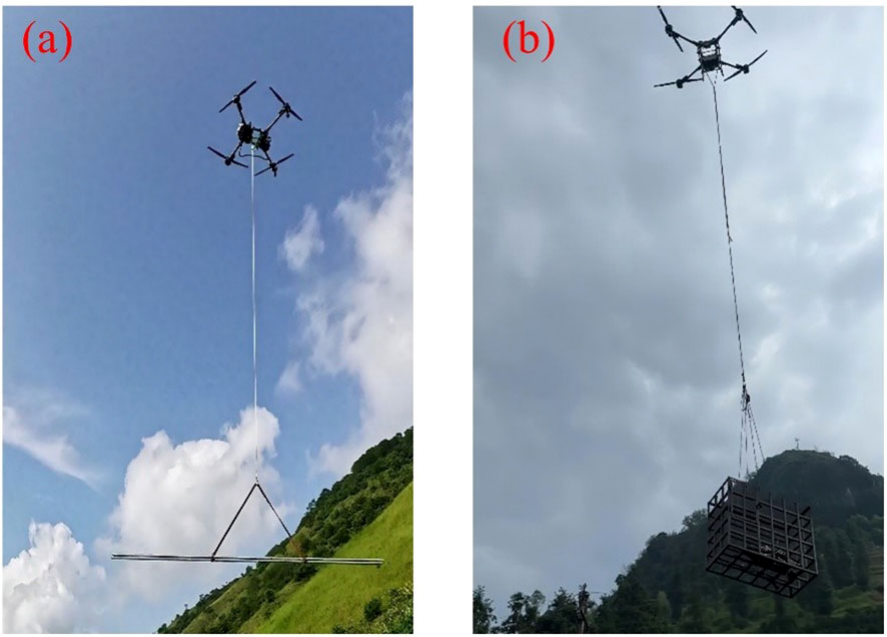
UAV applications in industrial logistics. **(a)** Transport of signal tower components, **(b)** Transport of sand, gravel, and cement.

Currently, logistics and transport still rely primarily on traditional manual delivery, which is not only inefficient and labor-intensive but also prone to causing damage to items. Although autonomous delivery vehicles have been applied in some scenarios, their use is restricted to specific locations and does not fundamentally address the challenges of logistics operations. The emergence of UPT and its gradual application in logistics provides a novel solution to these limitations, as illustrated in **Figure 17**: transporting signal tower components (**Figure 17a**) and hauling sand, gravel, and cement (**Figure 17b**).

Existing studies have explored various types of grasping mechanisms and transport structures to enhance UAV operational capability and environmental adaptability in logistics scenarios.

For example, Zhong et al. (2020) combined a single camera with ArUco markers and a Kalman filter, achieving notable improvements in UAV positioning accuracy during indoor material handling, with errors maintained within 8 cm, thereby providing technical support for precise operations in warehouse environments. Regarding structural optimization, Yang (2024) designed a carbon-fiber transport pod that integrates landing gear functionality with lateral conveyance capability, allowing UAVs to pick up and deposit items without landing, thus enhancing adaptability in space-constrained settings. To address the high weight and energy demands of conventional grippers. Hingston et al. (2020) developed two mechanisms-a mesh-based structure and a large slider-style parallel claw-both demonstrating effective performance in handling everyday objects while minimizing the payload’s impact on flight endurance. Furthermore, Wang et al. (2025) mounted a flexible spiral soft robot (SpiRobots) on a UAV platform, achieving agile grasping of objects of varying sizes and shapes using only 2–3 cables, with a load capacity up to 260 times its own weight, highlighting its potential for handling complex objects in practical applications.

In terms of safety and convenience, Kornatowski et al. (2017) integrated an origami-inspired foldable protective cage with a UAV platform, providing collision protection during cargo transport and allowing efficient post-flight storage, thereby substantially enhancing system portability and safety. To improve transport efficiency, Dukkanci et al. (2021) developed a delivery path model constrained by flight range, employing a perspective cutting method to simultaneously optimize UAV speed and energy consumption. Torabbeigi et al. (2019) further incorporated the effect of payload on battery usage into the scheduling model, demonstrating that neglecting this factor can render many paths infeasible, which underscores the importance of considering load-endurance coupling in task allocation. Additionally, biomimetic designs have emerged as a promising approach to enhance UAV grasping performance. Zhao et al. (2023), inspired by the structure of eagle legs, designed a bionic mechanical gripper suitable for medium- and large-sized multirotor UAVs, providing an efficient and reliable solution for contact-based operations. Building on this, Zhao et al. (2024b) developed a biomimetic leg-claw mechanism with active-passive grasping functionality, improving UAV interaction with the environment. These biomimetic grippers enable precise and safe object capture and release, offering effective support for UAV-based material transport.

Currently, UPT has demonstrated preliminary effectiveness in factory logistics and short- to medium-range cargo transport. Research has made progress in areas such as gripper structure optimization, path planning, and endurance management. However, several challenges remain in practical applications. For instance, the diversity of cargo shapes means that existing grippers still lack sufficient versatility and stability; in complex environments, variable obstacles and lighting conditions can affect target recognition and obstacle avoidance accuracy; additionally, limited battery endurance constrains long-duration or large-scale transport tasks. Future research could focus on three directions: first, designing modular and rapidly replaceable multifunctional grippers to accommodate cargo of varying sizes, weights, and materials; second, integrating multi-sensor fusion and machine vision algorithms to enhance obstacle detection and dynamic avoidance in complex environments; third, adopting high-efficiency batteries, wireless charging, or ground-assisted battery replacement mechanisms, while exploring multi-UAV cooperative transport strategies to improve overall system transport capacity and operational continuity.

#### 5.1.3 Engineering surveying

**Figure 18:**
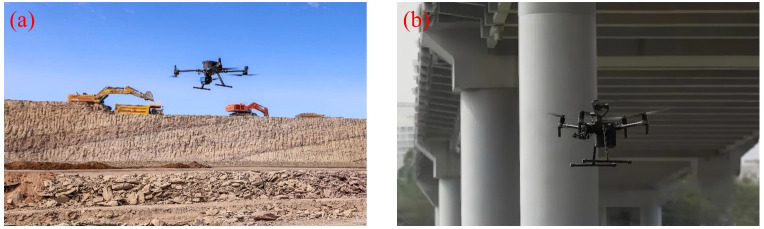
UAV applications in engineering surveying. **(a)** Mining area surveying, **(b)** Bridge inspection.

Traditional engineering surveying relies primarily on manual operations, which are not only inefficient and costly but also pose significant safety risks in complex environments such as mountainous regions, construction sites, and mining areas. UAVs, equipped with various sensors, have gradually emerged as a novel solution for road design, engineering surveying, and bridge inspection, significantly enhancing both efficiency and accuracy, as illustrated in **Figure 18**: UAV-based mining area surveying (**Figure 18a**) and UAV bridge inspection (**Figure 18b**).

In practical applications, Feng (2018) explored the workflow of UAV remote sensing for engineering projects, including flight path planning, image acquisition, and data processing, demonstrating its capability to obtain high-quality survey data in harsh environments and effectively enhancing both efficiency and safety. Lee et al. (2022) conducted earthwork measurement experiments in a highway construction scenario, showing that measurement errors were controlled within 2.36%-2.51% and that efficiency increased by over 30% after multiple operations, indicating its feasibility as a replacement for traditional methods. Zulkipli and Tahar (2018) utilized a multirotor UAV to collect terrain data required for road design, achieving excellent performance in both data accuracy and cost control, providing a practical approach for conventional surveying.

Moreover, UAVs are increasingly recognized for their potential in high-precision surveying. Bo et al. (2024) employed close-range aerial imaging to capture high-resolution images of building facades and constructed millimeter-level models, successfully enabling the identification and assessment of facade damage, thereby providing precise data support for urban renewal and building management. Van et al. (2023) integrated UAV aerial imagery with terrestrial LiDAR data to develop a three-dimensional mining area model compliant with CityGML standards, supporting digital twin applications in mine management. For bridge health monitoring, Gadiraju et al. (2025) explored a deep reinforcement learning-based approach for bridge deck crack detection, deploying a UAV on bridges with active traffic. The results demonstrated that the CNN-based method achieved significantly higher detection accuracy than traditional edge detection techniques, offering an efficient and intelligent solution for bridge structural maintenance.

The aforementioned studies indicate that UPT has demonstrated promising applications in engineering surveying, particularly in earthwork measurement, building facade modeling, and three-dimensional reconstruction of mining areas, enhancing both operational efficiency and data accuracy. However, existing research largely focuses on open areas with simple structures, and significant limitations remain in densely built, heavily occluded, or spatially constrained environments, especially regarding obstacle avoidance, flight path planning, and the accuracy of multi-sensor data fusion. Future research could focus on improving autonomous path planning and obstacle avoidance in complex urban areas, enhancing the fusion accuracy of multimodal sensors such as LiDAR, oblique photogrammetry, and visual SLAM for more precise and detailed modeling. Additionally, integrating deep learning with intelligent scheduling strategies can promote the unified development of mission planning, data processing, and 3D modeling, gradually realizing more efficient and intelligent surveying operations.

#### 5.1.4 Military

**Figure 19:**
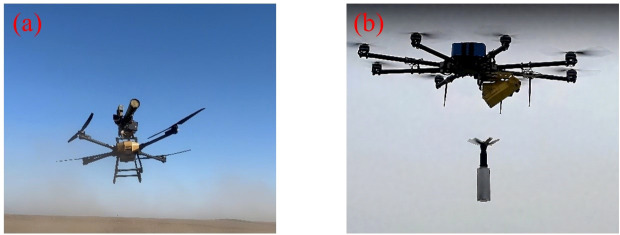
Military applications of UAVs. **(a)** firearm mounting, **(b)** UAV bomb deployment.

The evolving international security landscape, coupled with the rapid development of UPT, has led to its increasing application in military domains, as illustrated in **Figure 19a** (firearm mounting) and **Figure 19b** (UAV bomb deployment). In explosive ordnance disposal (EOD), Fan et al. (2021) employed a hexacopter UAV system equipped with a mechanical claw and winch, integrated with the YOLOv5 algorithm to identify and handle unexploded ordnance. This system enabled integrated operations of grasping, transferring, and neutralizing, significantly enhancing operational safety and flexibility. Lee et al. (2016), addressing military logistics support, designed a modular EOD gripper with an improved dual-mode torsion drive, offering lightweight construction and high force output, thereby improving operational precision and practicality of EOD equipment. Xin et al. (2023) and Chen (2021) examined multirotor UAVs across various applications, including supply delivery, equipment maintenance, and casualty evacuation, highlighting their efficiency and feasibility in challenging terrains. They emphasized advantages such as flexible deployment, strong stealth, and relatively low cost, while noting that endurance and resistance to interference still require improvement. To enhance task allocation efficiency, Schwarzrock et al. (2018) proposed an improved multi-UAV task assignment method based on swarm intelligence, which effectively increased task completion quality and system coordination in distributed environments. In the domain of intelligent reconnaissance, Lee et al. (2024) integrated target detection with reinforcement learning, enabling military UAVs to achieve higher recognition accuracy through joint training on image and vector data, illustrating a viable approach for augmenting autonomous reconnaissance capabilities. Furthermore, Cao and Sharma (2022) addressed communication coordination by applying reinforcement learning to optimize multi-UAV networks, thereby reducing information update delays and supporting efficient collaborative operations in complex battlefield scenarios.

UPT exhibits high safety and operational efficiency in military applications, including explosive ordnance disposal, reconnaissance, and logistical support. Nevertheless, several challenges remain, such as limited payload capacity, short endurance, and unstable performance in complex environments. Future research is likely to focus on increasing battery energy density and enabling rapid battery replacement, optimizing multi-UAV cooperative control algorithms, enhancing the accuracy of multi-sensor data fusion, and improving target recognition and localization under challenging conditions. Moreover, exploring modular payload designs could facilitate rapid switching between tasks and enable more efficient mission execution.

In summary, existing studies indicate that UPT has extensive applications in the industrial sector. In power inspection, they enable image acquisition, path planning, and contact-based operations; in logistics and transportation, they optimize gripping structures, plan transport routes, and manage endurance; in engineering surveying, they facilitate high-precision modeling and data collection; and in military tasks, they can be used for explosive ordnance disposal, reconnaissance, and material delivery. Overall, UAVs demonstrate highly efficient, flexible, and intelligent operational capabilities.

### 5.2 Public services

UPT has been steadily advancing, and its applications in public service sectors are becoming increasingly widespread. They now serve practical roles in areas such as medical supply delivery (Amicone et al., 2021), aerial cinematography (Montes-Romero et al., 2020), traffic monitoring (Sagar and Kumar, 2020), food delivery (Frachtenberg, 2019), courier logistics (Filiopoulou et al., 2025), and emergency response (Półka et al., 2018). This section reviews their use across three main domains: emergency and medical transport, urban management and cultural services, and urban logistics and delivery. By examining specific cases, it highlights payload configurations and real-world applications, while briefly addressing current challenges and potential directions for future development.

#### 5.2.1 Emergency response and medical transportation

**Figure 20:**
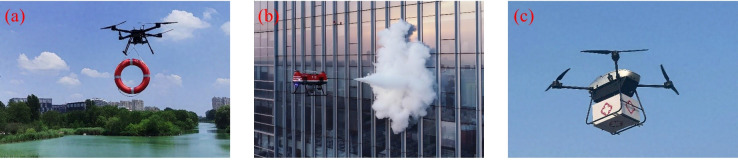
UAV applications in emergency response and medical scenarios. **(a)** Water rescue, **(b)** Firefighting, **(c)** Medical supplies transportation.

In scenarios such as sudden natural disasters and medical emergencies, UPT play an increasingly important role in enhancing response efficiency, owing to their rapid deployment, operational flexibility, and remote sensing capabilities. As shown in **Figure 20**, these technologies have been applied in water rescue, fire response, and medical supply transportation. In fire emergency management, Akhloufi et al. (2021) integrated sensing, perception, and cooperative technologies to study wildfire detection, monitoring, and firefighting support, achieving efficient fire information acquisition and optimized deployment. Mahesh et al. (2023) developed a firefighting UAV equipped with a mechanical gripper, which delivered ammonium phosphate fire suppression balls into high-risk areas and conducted targeted extinguishing operations, thereby improving operational efficiency while substantially reducing personnel risk. To enhance autonomous operation, Sandino et al. (2020) proposed a vision-based UAV autonomous navigation framework, which improved the intelligence level of operations at disaster sites.

Building on this, UPT is becoming increasingly mature in a variety of sudden disaster scenarios, such as landslides (Turner et al., 2015), earthquakes (Niethammer et al., 2012), and avalanches (Bejiga et al., 2017). Through rapid aerial surveying and image acquisition, it provides critical support for post-disaster assessment and operational planning. In medical transportation, Scalea et al. (2021) successfully conducted the field transport and transplantation of a human kidney, demonstrating the feasibility of UAVs for time-critical medical tasks. Braun et al. (2019) emphasized their practical value in pre-hospital emergency care, battlefield medicine, and the delivery of blood and pharmaceuticals, effectively reducing response times and expanding coverage.

To address the challenges of medical services in remote areas, Dixit et al. (2024) conducted a field test of UAV transport for sputum samples in Nepal, finding that it significantly improves sample connectivity and transport accessibility and is widely accepted by both medical institutions and local communities. Liu et al. (2024b) developed a truck-UAV collaborative delivery model and validated it in Guang’an, Sichuan, China. Their results indicated that the integrated system reduced costs by over 36.84% compared with single-mode delivery, and they suggested that further improvements in UAV endurance and charging efficiency could enhance overall operational performance. Additionally, Shi et al. (2022) proposed a bi-objective, multi-trip delivery optimization model. By applying an improved NSGA-II algorithm, their approach increased the efficiency of simultaneous pickup and delivery of medical supplies across multiple locations, offering a practical solution for logistics system design in public health emergency scenarios.

The role of UAVs in emergency response and medical transportation has gradually evolved from basic tasks, such as image capture and cargo delivery, to more integrated functions that include autonomous navigation, environmental sensing, and coordination across multiple platforms. Despite significant technological advances, limitations remain in complex weather adaptability, endurance, target recognition accuracy, and multi-UAV coordination efficiency. Future research may focus on several directions: first, integrating multi-source sensor data such as LiDAR and thermal imaging to enhance path recognition and obstacle avoidance under challenging conditions like smoke and nighttime; second, optimizing battery thermal management and rapid-swap designs to improve endurance and deployment efficiency according to mission cycles; third, promoting the development of air-ground information linkage mechanisms to enable dynamic task coordination and data sharing between UAV and ground rescue units, thereby enhancing overall response effectiveness.

#### 5.2.2 Urban management and cultural applications

**Figure 21:**
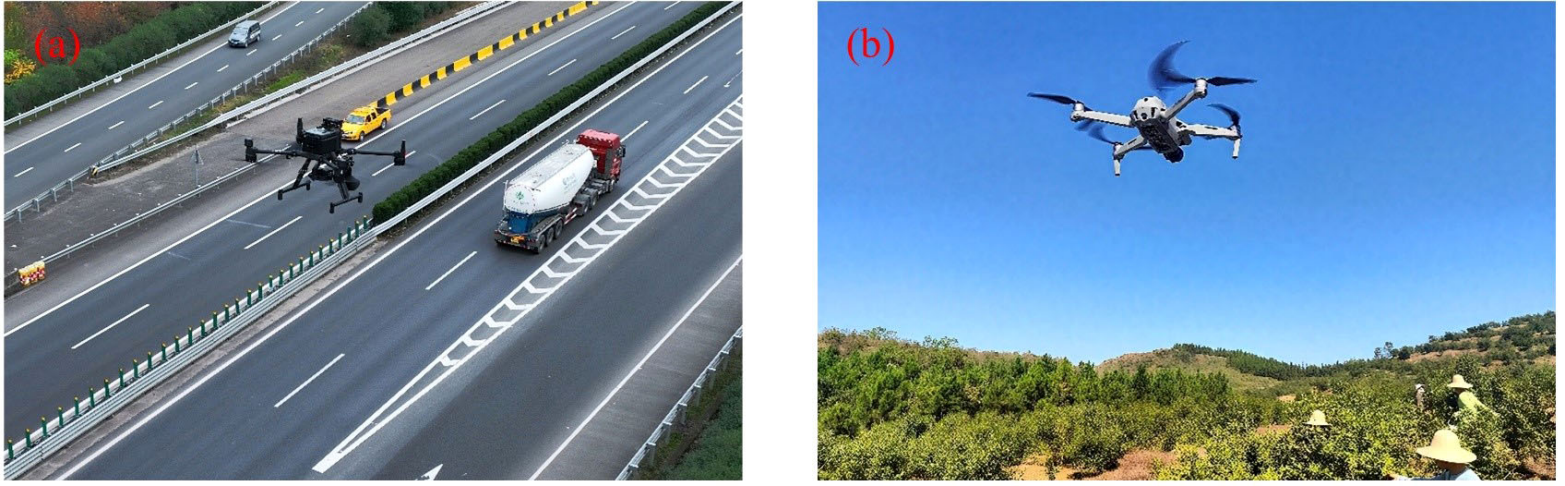
UAV applications in urban management and cultural scenarios. **(a)** Traffic management, **(b)** Aerial cinematography UAV (shooting a camellia oil tea field).

The application of UPT in urban traffic management and aerial cinematography is gradually increasing, demonstrating considerable practicality and development potential. As shown in **Figure 21**, UAVs are used for traffic supervision and film shooting. In traffic monitoring, Kumar et al. (2021) developed a software-defined UAV network-based surveillance system, which combined collision-avoidance strategies to enhance monitoring coverage and reduce communication energy consumption, providing an effective approach for data collection and scheduling in urban traffic scenarios. Bisio et al. (2022) reviewed UAV-based traffic monitoring systems and noted that deep learning techniques perform effectively in vehicle detection, tracking, and counting. Models trained on customized datasets achieved higher accuracy, underscoring the key role of data quality in system performance. In a similar study, Vohra et al. (2022) combined the YOLOv4 algorithm with UAV imagery to enable high-precision real-time vehicle recognition. Their system could automatically adjust traffic signal timings, thereby improving traffic flow and reducing accident risk, offering a novel technical solution for urban traffic management. In the domain of aerial cinematography, Ashtari et al. (2020) developed a UAV system that emulates the motion logic of human camera operators, allowing for smooth first-person perspectives and automatic transitions between different shot styles. Experiments demonstrated that the footage produced was comparable to manual operation in terms of style and stability, highlighting both the artistic potential and practical flexibility of UAV in film production.

Currently, UAVs in traffic monitoring possess capabilities for real-time detection and data transmission; however, their image quality and recognition accuracy are limited under low-light or adverse weather conditions, and densely built-up areas often cause communication interruptions, affecting system stability. In aerial cinematography, UAVs still rely on manually preset trajectories, making it difficult to dynamically adjust paths according to changes in scenes or storylines. Previous studies have demonstrated that infrared-visible light fusion algorithms can improve night-time recognition performance, while multi-channel or backup communication links enhance robustness. Future research can focus on integrating scene recognition with language-based analysis to achieve intelligent planning and dynamic adjustment of camera trajectories.

#### 5.2.3 Urban logistics and delivery

**Figure 22:**
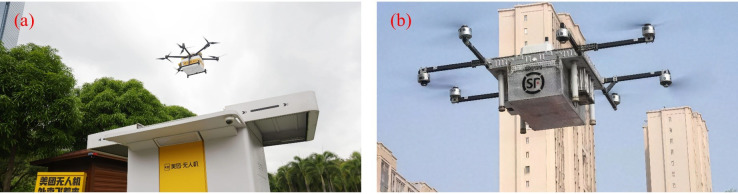
UAV applications in urban logistics. **(a)** Food delivery, **(b)** Courier services.

In urban food delivery and courier services, the introduction of UPT is gradually transforming traditional delivery models (**Figure 22**). In food delivery, Lu et al. (2024a) proposed a multi-distribution-center coordinated “UAV-rider” joint delivery model. They conducted an empirical analysis using Ele.me (The food delivery application developed by Shanghai Lazars Information Technology Co., Ltd., China) and Meituan food delivery (The food delivery application developed by Beijing Sankuai Online Technology Co., Ltd., China) orders, employing a two-stage heuristic algorithm (Euclidean distance-based order clustering and improved tabu search for route optimization). The results showed that this model effectively reduces the number of riders, lowers operational costs, and enhances customer satisfaction. Kwasiborska et al. (2023) compared various UAV and electric scooters in urban food delivery from the perspective of energy consumption, finding that small quadrotor UAVs consumed less energy in most scenarios, highlighting their potential for low-carbon transportation. Li et al. (2025d) used a multi-agent simulation model to evaluate the life-cycle emissions of UAVs and electric bicycles, indicating that although UAVs exhibit slightly higher emissions during the usage phase, they offer better overall economic performance. Furthermore, the use of clean energy can substantially reduce carbon emissions, providing a basis for green urban logistics.

In courier logistics, UAVs similarly demonstrate diverse technological approaches and system advantages. Wen and Wu (2022) proposed a scheme in which a large UAV carries a smaller UAV to achieve distributed delivery, designing a three-stage iterative algorithm to optimize overall route planning, which significantly improved efficiency and reduced costs. Zou et al. (2023) developed a locker-UAV collaborative system, combining package transfer and last-mile delivery optimization; multi-capacity UAV configurations reduced hardware investment while enhancing delivery efficiency. To improve navigation accuracy, Miranda et al. (2021) built a positioning system integrating GPS, IMU, ultra-wideband, and ArUco markers and employed vector field control to enhance autonomous landing stability. Suarez et al. (2024) designed an indoor air-drop system for special delivery needs, in which multirotor UAVs and compliant robotic arms collaboratively deliver packages directly from windows to users, increasing adaptability and human-robot safety in home deliveries. Kirschstein (2020) analyzed energy consumption differences between UAV and conventional trucks across various delivery scenarios, noting that UAVs offer no significant energy efficiency advantage in dense urban areas but are more competitive in suburban and rural settings. Tsykunov et al. (2021) further proposed DroneStick, a control interface composed of a UAV, a spool, and a vibration motor; its mechanical spooling and force-feedback mechanisms provide novel approaches for human-UAV interaction in delivery and control tasks.

Although current research has achieved preliminary results in route optimization, platform coordination, and last-mile delivery, UAVs still face challenges in navigation accuracy, energy efficiency, and delivery stability in complex urban environments. Future work can further integrate multi-source positioning technologies, such as BDS/GPS, vision, and ultra-wideband, to enhance navigation and obstacle avoidance in densely obstructed areas. Additionally, constructing energy management models under multi-UAV scheduling can improve the stability and endurance of coordinated operations. Moreover, improving end-point interaction devices for different residential structures may further enhance the adaptability and practicality of UAV payload systems.

## 6 Conclusions and outlook

This study reviews the development status and technological pathways of UAV payloads in agriculture and other typical application scenarios. It focuses on the performance of different types of sensors (such as multispectral, hyperspectral, and LiDAR), actuators (such as spraying systems, grasping mechanisms, and aerial lifting modules), and intelligent control algorithms in terms of operational efficiency, precision, and environmental adaptability. The research indicates that, with the continuous advancement of these technologies, UAV payloads have gradually formed an integrated “sky-to-ground” agricultural operation system covering sowing, fertilization, pest and disease control, and crop monitoring. For instance, the combination of multispectral and hyperspectral sensors has enhanced the ability to monitor crop growth and predict yields, while spraying systems coupled with variable-rate control modules have achieved more precise fertilization and pesticide application. Overall, UPT is driving agricultural operations toward greater intelligence and precision.

Although UPT has demonstrated promising applications in agricultural scenarios, its autonomous perception and path-planning capabilities remain limited in complex field environments. The payload platforms exhibit low versatility, and the lack of standardized interfaces between devices hinders seamless integration. Moreover, issues related to battery endurance, long-range communication, and operational stability under adverse weather conditions remain prominent. In addition, the overall systems still rely heavily on manual operation, which constrains the large-scale deployment and further advancement of intelligent agricultural operations.

Future research should further focus on the design of lightweight and multifunctional integrated payload systems, aiming to enhance platform endurance and carrying capacity through optimized materials and structural design. In terms of intelligent operations, multi-source sensor data fusion should be strengthened and combined with crop growth models and environmental parameters to achieve precise operational control. At the same time, modular and standardized integration between task payloads and UAV platforms should be promoted to improve rapid task switching and cross-platform adaptability. At the system level, a “satellite-UAV-ground” collaborative sensing and decision-making mechanism could be established, and the application of AI algorithms in crop condition recognition, operational decision-making, and path planning could be enhanced, thereby effectively advancing the unmanned and intelligent development of agriculture.

With the rapid development of the low-altitude economy and the modernization of agriculture, UPT is expected to continue expanding in the agricultural sector and extend to industrial, military, and urban service applications. It plays a significant role in transforming operational modes, enhancing production efficiency, and generating new types of job opportunities, while also providing new prospects for cultivating technical talent and promoting high-quality development across related industries.
