# Correction: Unmanned aerial vehicle payload technology applications in agriculture and other low altitude scenarios: a review

**DOI:** 10.3389/fpls.2025.1766846

**Published:** 2026-01-05

**Authors:** Weixiang Yao, Changliang Liu, Yuzhou Liu, Qi Zheng, Junyong Wang, Huiduo Yu, Chunling Chen, Shuang Guo

**Affiliations:** 1 College of Information and Electrical Engineering, Shenyang Agricultural University, National Digital Agriculture Regional Innovation Center (Northeast), Shenyang, China; 2 School of Intelligent Science and Information Engineering, Shenyang University, Shenyang, China

**Keywords:** agriculture, UAV, payload technology, application scenarios, low-altitude economy

An incorrect funding number, “2024YFD15011503” was provided for the **Funding** statement as published. The correct number is “2024YFD1501503”.

The original version of this article has been updated.

